# Enigmatic Evolutionary History of Porphobilinogen Deaminase in Eukaryotic Phototrophs

**DOI:** 10.3390/biology10050386

**Published:** 2021-04-29

**Authors:** Miroslav Oborník

**Affiliations:** 1 Biology Centre CAS, Institute of Parasitology, Branišovská 31, 370 05 České Budějovice, Czech Republic; obornik@paru.cas.cz; 2 Faculty of Science, University of South Bohemia, Branišovská 31, 370 05 České Budějovice, Czech Republic

**Keywords:** porphobilinogen deaminase, hydroxymethylbilane synthase, heme biosynthesis, evolution, mitochondrion, gene replacement, horizontal gene transfer

## Abstract

**Simple Summary:**

The heme pathway is essential for most of cellular life. In eukaryotic phototrophs, the entire pathway is plastid localized. Despite that, the enzyme responsible for the synthesis of hydroxymethylbilane, porphobilinogen deaminase, shows α-proteobacterial instead of expected cyanobacterial origins in rhodophytes, chlorophytes, plants, and most algae with complex plastid. However, no such enzyme has been found in the supposed partners of plastid endosymbioses, the heterotrophic eukaryotes, and cyanobacteria. I propose two scenarios explaining this phenomenon by either endosymbiotic gene transfer from the ancestor of mitochondria or a non-endosymbiotic lateral gene transfer from unspecified α-proteobacterium. Phylogenetic analysis of porphobilinogen deaminases does not reject any of the two proposed evolutionary scenarios.

**Abstract:**

In most eukaryotic phototrophs, the entire heme synthesis is localized to the plastid, and enzymes of cyanobacterial origin dominate the pathway. Despite that, porphobilinogen deaminase (PBGD), the enzyme responsible for the synthesis of hydroxymethybilane in the plastid, shows phylogenetic affiliation to α-proteobacteria, the supposed ancestor of mitochondria. Surprisingly, no PBGD of such origin is found in the heme pathway of the supposed partners of the primary plastid endosymbiosis, a primarily heterotrophic eukaryote, and a cyanobacterium. It appears that α-proteobacterial PBGD is absent from glaucophytes but is present in rhodophytes, chlorophytes, plants, and most algae with complex plastids. This may suggest that in eukaryotic phototrophs, except for glaucophytes, either the gene from the mitochondrial ancestor was retained while the cyanobacterial and eukaryotic pseudoparalogs were lost in evolution, or the gene was acquired by non-endosymbiotic gene transfer from an unspecified α-proteobacterium and functionally replaced its cyanobacterial and eukaryotic counterparts.

## 1. Introduction

The heme pathway is one of the metabolic routes essential for life as we know it. So far, we know of only a few organisms—the kinetoplastid parasite of plants, *Phytomonas serpens*, and several bacteria—that can live without heme [1]. It has been shown that the enzymatic composition of the pathway and the origins of the enzymes of heme (tetrapyrrole) biosynthesis have been deeply influenced by past endosymbiotic events [2,3,4,5,6]. This effect is visible in the pathway’s differing compartmentalization: To the mitochondrion and the cytosol in eukaryotic heterotrophs, to the plastid in phototrophic eukaryotes, and to rare combinations of the aforementioned localizations in apicomplexan parasites [7], chromerids [8], colpodellids [9], and the early-branching heterotrophic rhodophyte-like Rhodelphidia [10]. Likewise, the mosaic origin of the pathway reflects the involvement of different symbiotic partners in the evolutionary history of eukaryotes [2,3,8]. In eukaryotes (except for *Paulinella chromatophora*), all the enzymes of the pathway are encoded in the nucleus, expressed in the cytosol, and post-translationally targeted to their places of action. In phototrophic eukaryotes, enzymes of the original exosymbiont (eukaryotic host) pathway have mostly been lost, and the pathway has almost entirely been replaced by the plastid-located endosymbiont-derived metabolic route [2,4,5].

Porphobilinogen deaminase (PBGD) (syn. hydroxymethylbilane synthase) is the enzyme that catalyzes the third (eukaryotic heterotrophs) or fourth (eukaryotic phototrophs) step of the heme biosynthesis pathway. It converts porphobilinogen to hydroxymethylbilane, and this reaction is located in the cytosol of heterotrophs or the plastid of eukaryotic phototrophs, apicomplexan parasites [2], and *Rodelphis* [10]. Plants, chlorophytes, rhodophytes, and algae with complex plastids do not use PBGD of cyanobacterial origin as would be expected for this plastid-located enzyme and contrary to most of the other enzymes involved in heme biosynthesis (e.g., glutamyl-tRNA reductase (GTR), glutamate-1-semialdehyde 2,1-aminomutase (GSA/AT), aminolevulinate dehydratase (ALAD), uroporphyrinogen dehydratase (UROD), protoporphyrinogen oxidase (PPOX) and ferrochelatase (FeCH)). Instead, their PBGD strongly affiliates to α-proteobacteria [2,4,5]. The only exceptions that I have found are: A likely cyanobacterial PBGD in the glaucophyte *C. paradoxa* (this work), a γ-proteobacterial enzyme in the euglenophyte *Euglena gracilis* [3], an enzyme of unspecified origin in the green dinoflagellate *L. chlorophorum* [4], and the PBGD encoded in the genome of the cyanobacterial endosymbiont of the cercozoan primary phototroph *P. chromatophora* (this work).

A thorough inspection of the origins of PBGDs in the participants of plastid endosymbioses, particularly the heterotrophic eukaryotic exosymbiont (primary host) and cyanobacterial endosymbiont, found no gene encoding PBGD of α-proteobacterial origin. The enzyme from the primary host originates in an ancient eukaryotic nucleus [2]. However, all plants, chlorophytes and rhodophytes, and most algae with secondary plastids contain PBGD of α-proteobacterial origin. The logical question arises: Where did the α-proteobacterial enzyme come from?

## 2. Materials and Methods

The amino acid sequences of PBGD from various organisms were downloaded from GenBank^TM^ and aligned using Muscle [11] in SeaView [12]. The alignment was trimmed by Gblocs implemented in SeaView [12] and manually edited in BioEdit [13] (Supplementary Materials 1). Modelgenerator v. 0.85 [14] favored the LG+I+Γ model for maximum likelihood (ML) phylogenetic analysis, and the ML tree was constructed with this model using RAxML v. 8.2.12 [15] with 200 bootstraps. Bayesian analysis was performed with Phylobayes v. 4.1c (GTR+CAT model) [16], running two independent chains for 10,000 cycles. Chain convergence as well as effective sample size was checked with bpcomp and tracecomp tools from the Phylobayes package.

## 3. Results

**Figure 1:**
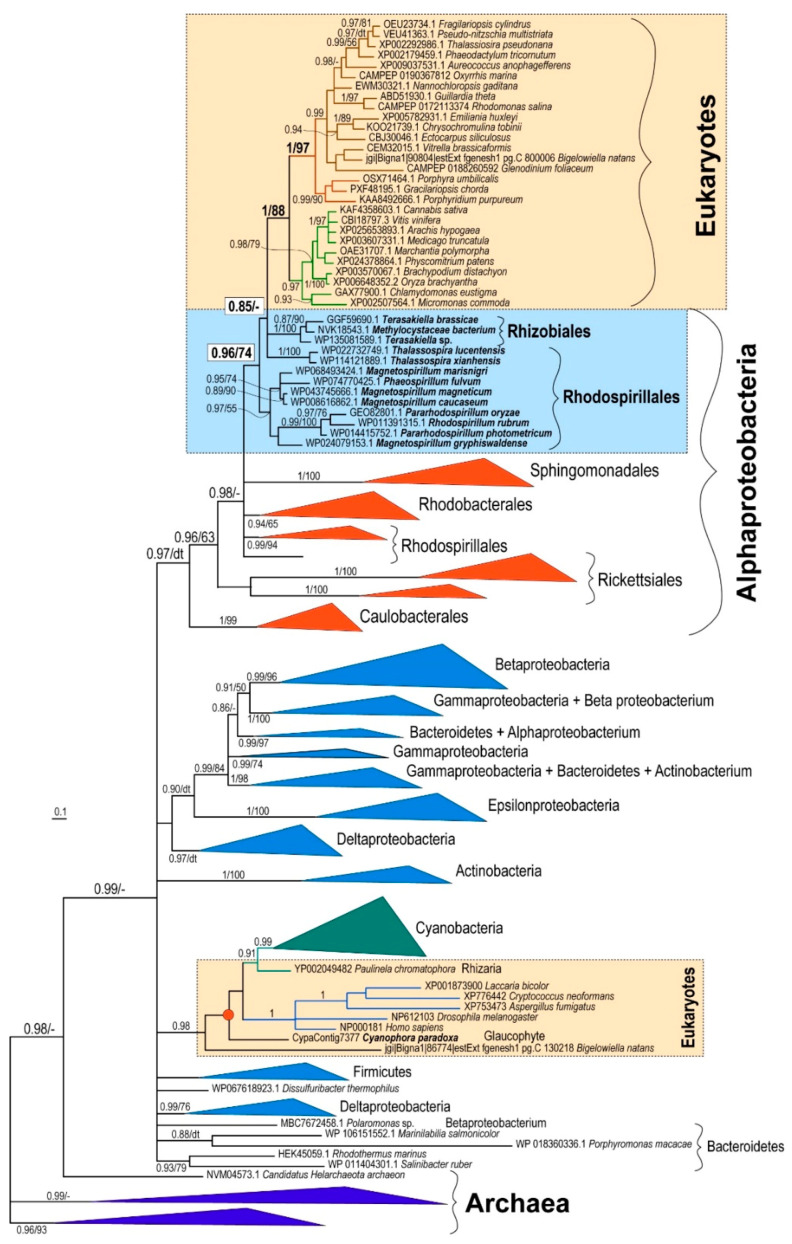
Schematic Bayesian phylogenetic tree as inferred from amino acid sequences of PBGD. Numbers above branches indicate Bayesian posterior probabilities/maximum-likelihood bootstrap supports. “dt” indicates different topology by maximum likelihood (see Figures S1 and S2 for details); “-” indicates the same but unsupported topology. Full trees are available in the Supplementary Materials.

To resolve the question concerning the origin of PBGD in eukaryotic phototrophs, I performed a phylogenetic analysis of PBGD amino acid sequences. I started with a large dataset containing more than 250 sequences from eukaryotes, bacteria, and archaea. However, as the gene is not long, after automated trimming, the alignment contained only 203 sites. Therefore, due to the limited information content of the gene, the backbone of the initial maximum likelihood tree was not resolved and the crucial basal nodes were not supported at all (not shown). With a reduced dataset of 169 OTUs, the backbones of the Bayesian [16] and maximum likelihood [15] trees were still not highly supported. Nevertheless, the different methods yielded similar topologies. In all of the PBGD trees, most of the phototrophic eukaryotes (rhodophytes, chlorophytes, plants, diatoms, cryptophytes, haptophytes, chromerids, dinoflagellates, pelagophytes, and eustigmatophytes) constituted a well-supported (posterior probability, pp, 1/bootstrap support, bs, 88) monophyletic clade (Figure 1, Figures S1 and S2). In both maximum likelihood and Bayesian trees, Rhizobiales and Rhodospirillales were the most closely related bacterial groups to the plastid-located PBGD enzymes. The position of Rhizobiales had very weak support (pp 0.85/bs 40). However, there was moderate support (pp 0.96/bs 74) for the grouping of eukaryotes with Rhizobiales and the Rhodospirillales subgroup (Figure 1, Figures S1 and S2). It is important to note that Rhodospirillales are, in contrast to previously published trees [17], polyphyletic in both PBGD trees (Figure 1, Figures S1 and S2).

## 4. Discussion

Seeking the origin of plastid-located PBGD in phototrophic eukaryotes requires an investigation of the endosymbiotic event preceding the acquisition of cyanobacteria, the supposed ancestor of primary plastids. It is evident that a eukaryotic cell participating in the plastid endosymbiosis in the role of an exosymbiont (primary host) passed, before the acquisition of the plastid, through the primary endosymbiotic event (or series of events) with a proteobacterium that subsequently evolved into the mitochondrion. Since heme is generally indispensable for the survival of any cell, participants in the “mitochondrial” endosymbiosis, a pre-eukaryotic cell and an α-proteobacterium, are presumed to have possessed their own tetrapyrrole biosynthetic routes. The first common precursor of the pathway, δ-aminolevulinate (ALA), can be synthesized by two different routes. The C4 pathway, synthesis of ALA by condensation of succinyl-CoA and glycine, catalyzed by ALA synthase, is present only in α-proteobacteria and the mitochondria of primary eukaryotic heterotrophs. All other organisms use the C5 pathway (synthesis of ALA from glutamate) as their ALA source [2,4,5]. We can easily look at the composition of the heme pathway in α-proteobacteria, but we can only speculate about heme synthesis in a hypothetical pre-eukaryotic mitochondrion-free cell. In particular, Archaea (ASGARD group [18]), a supposed representation of a pre-eukaryotic cell, may have used a somewhat different heme pathway as compared to other cells: It has been shown that Archaea and also denitrifying and sulfate-reducing bacteria possess modified heme synthesis, synthesizing heme and d1 heme from precorrin-2 (siroheme biosynthesis) [19,20]. At the same time, all prokaryotes except α-proteobacteria synthesize ALA by the C5 pathway. Since most of the heme pathway enzymes (including PBGD) in primary heterotrophic eukaryotes (e.g., animals, fungi, amoebozoans) appear to be of eukaryotic origin [2,4,5], and because the C4 pathway is rare and specific for a single bacterial group (α-proteobacteria), it is very likely that the pre-eukaryotic cell used the C5 pathway. For this reason and due to the absence of the mitochondrial TCA cycle (the source of succinyl-CoA in eukaryotes) in a pre-eukaryotic cell, we can assume that the host involved in the (primary) mitochondrial endosymbiosis possessed the heme route with the C5 pathway using glutamate as the starting substrate.

However, I should note here that no single enzyme involved in the heme pathway in eukaryotes shows direct phylogenetic affiliation to an archaeal counterpart [4]. It partially reflects the presence of the α-proteobacterial C4 pathway in heterotrophic eukaryotes and the entire cyanobacterial heme pathway in eukaryotic phototrophs. In eukaryotic heterotrophs, the remaining enzymes of the pathway constitute distinct monophyletic sister groups to unspecified bacterial homologs. Such genes were likely acquired by the pre-eukaryotic cell before adopting mitochondria, by the non-endosymbiotic LGT, or reflect hidden endosymbiotic events preceding the appearance of mitochondria.

**Figure 2:**
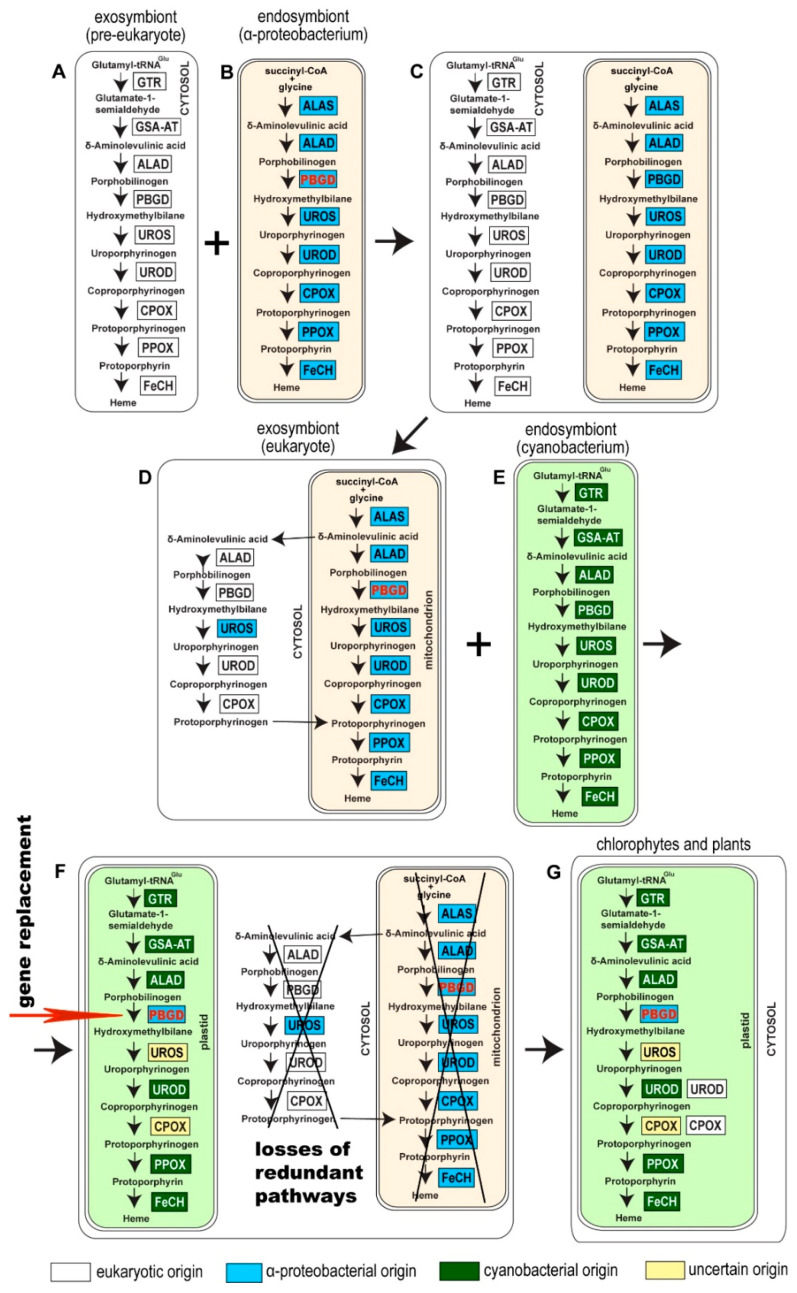
Evolutionary schema showing a possible scenario leading to the appearance of mitochondrial PBGD in phototrophic eukaryotes. A pre-eukaryotic cell (**A**) engulfed (or was invaded by) an α-proteobacterium (**B**). Two complete redundant heme biosynthesis pathways were present in an early eukaryotic cell after acquiring the ancestor of mitochondria (**C**). The origins of genes in a pre-eukaryotic cell are unclear. However, they are not of archaeal origins, and they rather came from various bacteria through LGT. The heme pathway became redundant after the endosymbiotic event, and both the host and endosymbiont pathways were reduced. In heterotrophic eukaryotes, the first step of the route (synthesis of ALA) is mitochondrial. The next four steps are located in the cytosol, and the path is terminated in the mitochondrion again. In the time of primary plastid endosymbiosis, the α-proteobacterial pathway was not fully reduced yet, and the mitochondrial PBGD was still present in the cell (**D**). When a heterotrophic eukaryote engulfed a cyanobacterium (**E**), the mitochondrially-cytosolic pathway was gradually lost, except for PBGD, which replaced the cyanobacterial homolog in the plastid (**F**). As a result of past endosymbiotic events, photosynthetic eukaryotes possess the plastid localized heme biosynthesis pathway composed of enzymes of various origins (**G**). All the genes in question were nuclear-encoded and posttranslationally to the place of action. Abbreviations of enzymes: GTR: glutamyl-tRNA reductase, GSA-AT: Glutamate-1-semialdehyde aminomutase, ALAS: ALA synthase, ALAD: ALA dehydratase, PBGD: Porphobilinogen deaminase, UROS: Uroporphyrinogen synthase, UROD: Uroporphyrinogen dehydratase, CPOX: Coproporphyrinogen oxidase, PPOX: Protoporphyrinogen oxidase, FeCH: Ferrochelatase.

**Figure 3:**
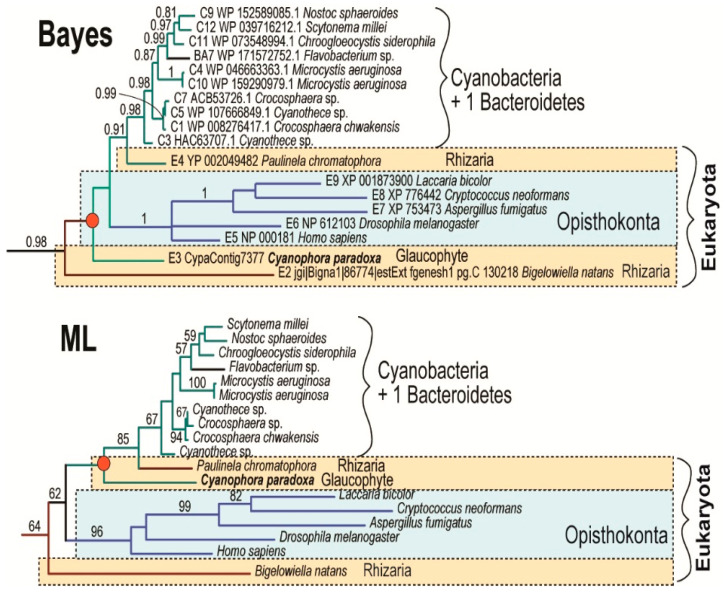
Alternative topologies are showing the phylogenetic positions of glaucophyte *C. paradoxa* as inferred by Bayesian and maximum-likelihood analyses. The phylogenetic position of the glaucophyte *C. paradoxa* is unstable. However, maximum likelihood analysis placed the glaucophyte on the root of the cyanobacterial clade. Numbers above branches indicate Bayesian posterior probabilities (Bayes) and maximum-likelihood bootstrap support (ML).

**Figure 4:**
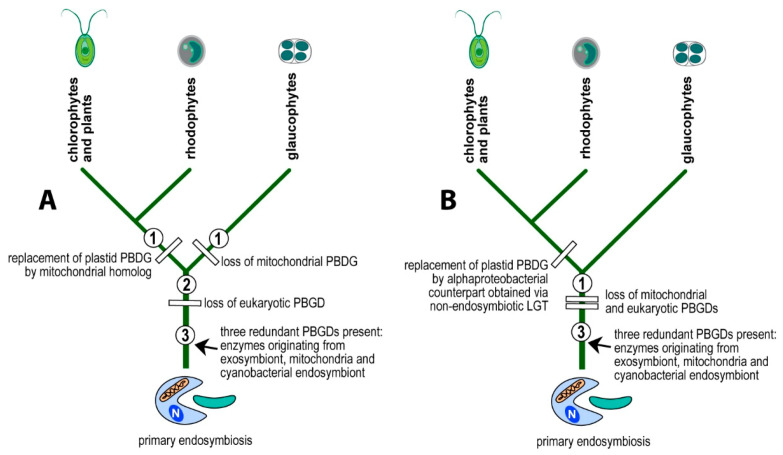
Alternative explanations of the evolution of PBGDs in primary eukaryotic phototrophs. The α-proteobacterial PBGD in eukaryotic phototrophs is either of the mitochondrial origin and had replaced the plastid nuclear-encoded gene followed by retargeting the enzyme to the plastid (**A**) or originates in the non-endosymbiotic LGT from an unspecified α-proteobacterium (**B**).

Acquisition of the α-proteobacterial endosymbiont presumably led to redundancy in heme biosynthesis in an early eukaryotic cell. It contained the cytosolic, exosymbiont originated pathway, presumably starting through C5 with glutamate as the initial substrate, in addition to the α-proteobacterial pathway in the endosymbiont, with the synthesis of ALA by C4 from glycine and succinyl-CoA (Figure 2C). The analogous presence of two redundant pathways for heme synthesis has already been proposed in the case of plastid endosymbioses: Secondary algae such as euglenophytes and chlorarachniophytes retain both the exosymbiont- and endosymbiont-derived heme pathways within a single cell [4,8]. A similar situation is expected in *Paulinella chromatophora*, which has the most recently acquired cyanobacterial endosymbiont, and still encodes 9 of the 10 enzymes involved in the autotrophic heme pathway in the “plastid” genome. Surprisingly, in the glaucophyte *C. paradoxa* only PBGD of likely cyanobacterial origin was found. The position of the glaucophyte gene in the tree was unstable when I constructed the tree by different methods (Figure 3). While maximum likelihood analysis placed the glaucophyte PBGD on the root of cyanobacterial counterparts, the Bayesian inference preferred its position on the root of the clade composed of cyanobacterial and opisthokonts genes. However, performed analyses did not support any of the alternative positions. Although we cannot be sure with the particular position of the glaucophyte in the tree (Figure 3), it is evident that their PBGD is not, in contrast to other eukaryotic phototrophs, of mitochondrial (α-proteobacterial) origin. This means that at least before the divergence of glaucophytes, two genes encoding PBGD (mitochondrial and cyanobacterial in origins) were almost certainly still present in the cell (Figure 2 and Figure 4).

I propose a scenario in which the eukaryotic PBGD was lost before the divergence of glaucophytes, rhodophytes, and chlorophytes. The mitochondrial pseudoparalog was replaced by the cyanobacterial homolog in the branch leading to glaucophytes (Figure 2 and Figure 4A). In the lineage leading to rhodophytes, chlorophytes, and plants, the mitochondrial PBGD was redirected to the plastid, replacing the plastid-originated enzyme in the pathway (Figure 2 and Figure 4) with the subsequent loss of the plastid-originated gene. Such a process was conditioned by the gradual losses of redundant heme pathways following endosymbiotic events. Therefore, at the time of acquiring a cyanobacterial endosymbiont, the PBGD of the mitochondrial origin was still present in the cell and was not yet replaced by the gene from a pre-eukaryotic cell (Figure 2) as it was in primary heterotrophic eukaryotes.

The second possible scenario interprets the presence of α-proteobacterial PBGD in eukaryotic phototrophs to be the result of a non-endosymbiotic transfer of the gene encoding PBGD from an α-proteobacterium (Figure 4B), instead of from an endosymbiotic gene transfer followed by the gradual loss of pseudoparalogs (exosymbiont, mitochondrial, cyanobacterial). To test the two alternative scenarios, I performed a phylogenetic analysis of PBGDs from various bacteria, Archaea, and eukaryotes (Figure 1). The genes from eukaryotic autotrophs (chlorophytes, plants, rhodophytes, and algae with complex plastids) invariably appear within the clade of α-proteobacteria, in a sister position to *Rhizobiales* and *Rhodospirillales*, as was previously shown on much smaller datasets [2,3,4,8]. Although it is generally accepted that mitochondria originate from α-proteobacterial endosymbionts, the phylogenetic position of mitochondria within α-proteobacteria is not clear. Most authors claim that mitochondria evolved from *Rickettsiales* or some related group of bacteria [21,22,23,24,25,26]. Another study [27] based on the analysis of 354 sequenced genomes (286 eubacterial, 24 archaeal, and 44 eukaryotic) found that *Rhizobiales* and *Rhodobacterales* have many proteins with affiliation to mitochondria. A phylogenomic analysis by Esser and colleagues [28] suggested that all tested α-proteobacteria (*Agrobacterium*, *Brucella*, *Mesorhizobium*, *Novosphingobium*, *Rhodobacter*, *Rhodopseudomonas*, *Rhodospirillum*, *Rickettsia*, *Wolbachia*) appeared equally related to mitochondria. In other words, some proteins of each α-proteobacterial lineage show close relationship to mitochondrial homologs. Similarly, work published by Abhishek et al. [29] demonstrated that mitochondrial genomes are evolutionary mosaics displaying phylogenetic affiliations to various α-proteobacterial groups, with the highest portion of genes related to *Rhodospirillum rubrum* and *Rhodopseudomonas palustris*. A recently published complex network approach [30] suggests that mitochondria share a common ancestor with a clade containing all α-proteobacterial orders, except *Rickettsiales*.

The latest phylogenomic analysis, which also involved bacterial metagenomic data from the TARA Ocean expedition, suggests that mitochondria evolved from a proteobacterial lineage that branched off before the divergence of the sampled α-proteobacteria [31]. In the trees I constructed, *Rhizobiales* and *Rhodospirillales* invariably represented the α-proteobacterial groups most closely related to the plastid-targeted PBGDs (Figure 1). According to several of the works mentioned above [27,28,29,30], this phylogenetic position would suggest a possible mitochondrial origin for this gene but it is not in agreement with studies suggesting that the origin of mitochondria is in *Rickettsiales* [21,22,23,24,25,26]. The robustness of these phylogenetic trees was not high, particularly the maximum-likelihood tree. However, the positions of α-proteobacterial subgroups were well supported by Bayesian posterior probabilities (pp 0.94–0.98). Importantly, the tree topology is consistent with previously published PBGD phylogenies [2,3,4,8].

Gene replacements and the selection that occurs among the population of pseudoparalogs following endosymbiotic acquisition of organelles are fascinating evolutionary phenomena. It is a clear example of genes “behaving” according to the selfish-gene hypothesis [32]. Since a redundancy of genes coding for the same protein is costly for the host cell, pseudoparalogous genes obtained during endosymbiotic processes compete like independent individuals for the host cell’s resources. It is apparent that the origin of the gene in the compartment where the protein locates is not always an advantage favoring the gene in the selection process. Some enzymes, such as ferrochelatase, have been frequently replaced in the evolution of phototrophic eukaryotes. This enzyme, which catalyzes the last step in heme biosynthesis, was independently replaced in rhodophytes, dinoflagellates, apicomplexan parasites, and euglenophytes. However, such replacements are very likely associated with the change of the regulatory mechanism of the pathway [33,34]. The only way for the gene to escape from this fratricidal struggle is to acquire a new function.

## 5. Conclusions

Although the heme biosynthesis pathway is plastid localized in eukaryotic phototrophs, one of the enzymes, porphobilinogen deaminase (PBGD), shows α-proteobacterial origin in rhodophytes, chlorophytes, plants, and most algae with complex plastids. This origin is unexpected because there was no α-proteobacterial PBGD in the supposed partners of plastid endosymbiosis. Phylogenetic analysis cannot reject the mitochondrial origin of the gene and confirm the alternative hypothesis. The gene was either transferred from an ancestor of mitochondria when multiple redundant heme pathways were present in the endosymbiotic cell assembly, or it was obtained in a non-endosymbiotic gene transfer from α-proteobacteria. A single group of primary eukaryotic phototrophs, glaucophytes, uses the cyanobacterial pseudoparalog of PBGD, suggesting that the gene replacement occurred after the divergence of glaucophytes.

## Data Availability

The phylogenetic dataset is a part of the Supplementary Materials.

## Supplementary Materials

The following are available online at https://www.mdpi.com/article/10.3390/biology10050386/s1, Figure S1: Bayesian phylogenetic tree as inferred from amino acid sequenced of PBGD, Figure S2: Maximum likelihood phylogenetic tree as inferred from amino acid sequences of PBGD, Dataset S1: Multiple alignment (fasta) of PBGD amino acid sequences used for the constructions of phylogenetic trees.
