# Supplementary material for: Enigmatic Evolutionary History of Porphobilinogen Deaminase in Eukaryotic Phototrophs
Source: Biology (Basel). 2021 Apr 29;10(5):386. doi: 10.3390/biology10050386 (PMC8145841; doi:10.3390/biology10050386)
Supplement: Supplementary file 1 [file biology-10-00386-s001.zip › biology-1177644-supplementary.pdf]

Supplementary material

Supplementary figure S1: Bayesian phylogenetic tree as inferred from aminoacid sequenced of PBGD. Numbers above brachnes indicate Bayesian posterior probabilities.

Supplementary figure S2: Maximum likelihood phylogenetic tree as inferred from amio acid sequences of PBGD. Numbers above branches indicate ML bootstrap support.

Dataset S1: Multiple alignment (fasta) of PBGD aminoacid sequences used for the constructions of phylogenetic trees.

Supplementary Figure 1:

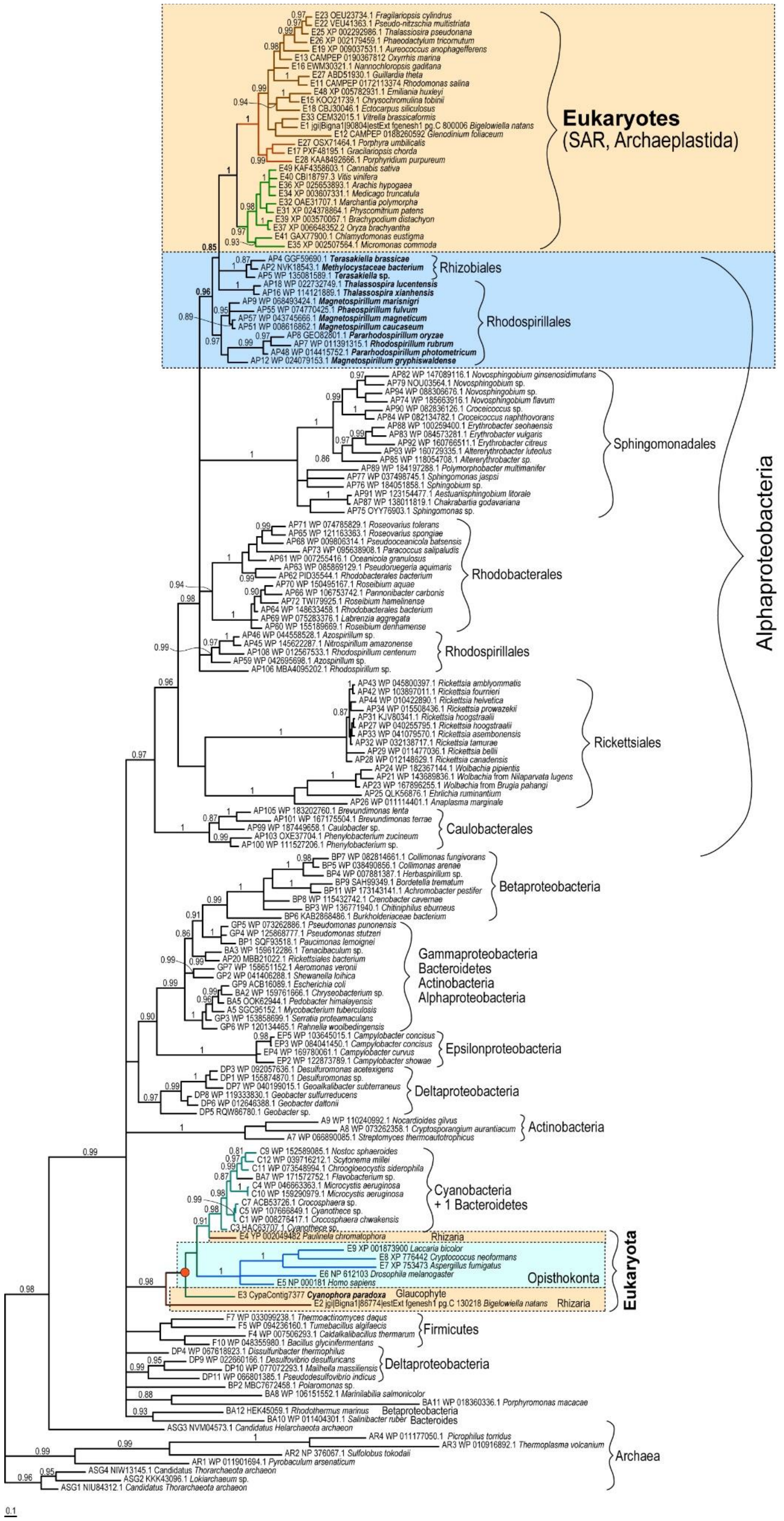

Supplementary figure 2:

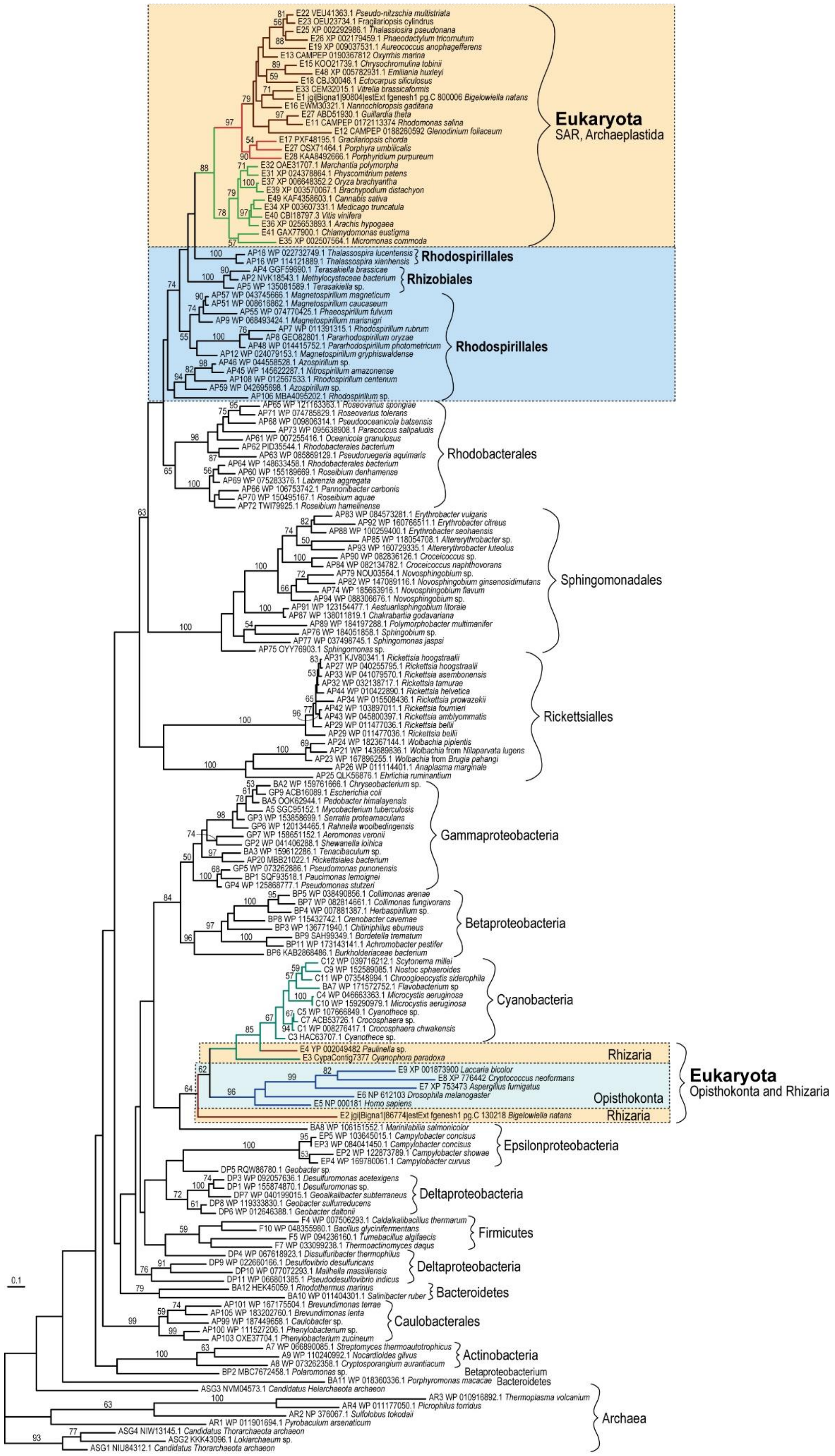

Supplementary file 1:

```
>AR1_WP_011901694.1_Pyrobaculum_arsenaticum
VVATRGSKLSMLQTEELLAQIEIKIVKTTGDEVQDKPLYVKGIFEKEVNLAVLRGEADVAVHSLKDLPSELSPGLVLAGFSKRAPPFDAVVSVL
ETLPRGALVGTSSVRRAEFVKAIRPDVRGNVDTRINKIDAAIMAVAGIYRLPPPGQGIVAAVVREADASDPKASLEAIAEREFLKAVGAGCHVA
IGGVAVEFIAPVEVGRRAAEEI
>AR2_NP_376067.1_Sulfolobus_tokodaii
RIAARGSKLSRIQVMMVENYLEFIEIKTKADLFQNEPLS-
KGVFEKEVNQAVLDNKADVAVHSMKDILTEISENLEIYAVLPRDPPFDILISNLFNLEPNSIIGTSSIRRKNFLTFYRNDLRGNIDTRLKKYDG
IIIAEASIYRLPEANQGIIVVIGRKKDINDKDTLEIAIAERKALSIVGGGCHSPIGVYFKDFHGGELLGKRFLEEI
>AR3_WP_010916892.1_Thermoplasma_volcanium
KIGTRPSNLAVAQANMVASSLEIVKHRSAGDIDTKNPIYKTGVFVQDLNNMILRGEIDVAVHSAKDIPSEIENRLTIAATLKRGSFNDALVSDL
RSLPISAKVGTSSIRRIFQLKYARQDLRGNIETRIAKMDAIVMAEAAIQRLPAPNQGIIAVVS-
KKEINDENTFEEMQIERSVVQQLKLGCSTPVGILSIIAQFVDSLVSYIRENI
>AR4_WP_011177050.1Picrophilus_torridus
RIGTRSSKLAMIQAMMVKDRLEVKGFTSKGDINLDSPLYGTGVFVDDLNRMILKNEIDIAVHSAKDIPSFIDDSLEISAVLKRDDPRDVLISSL
NDLEASSVIGTSSLRRIKELKTLRNDIRGNIDTRLKKLDGIIMAKAAYDRMPAPNQGIIAIIS-
KKDINDDETYNDMKAERLILSGLNLGCSKPVGIYAIFMRFYNNIDLEFIRSE
>ASG1_NIU84312.1_Candidatus_Thorarchaeota_archaeon
TVGTRGSKLSLIQTENVIQKLEVQVIKTIGDEVTSKPLIEKGIFEKEIDKAVLRGDVDIAVHSMKDLPTIRPHKTIILAVPERESLYDALVSRL
KALPNKAIIGTGSPRRKAQVLHMRPDIRGNVDTRVRKLDAIILAEAGLQRLPAPGQGALAIVARQDDINHWPSMASVLAERSFIKEIGGGCRVP
LGAFALSLHAPDELGFKVAQKI
>ASG2_KKK43096.1_Lokiarchaeum_sp
IVGTRGSKLSLVQTDNVIQKLNVKIIKTYGDRKSHKPLIERGVFVKSLDKAVLEGKVDFAVHSMKDVPTKMLPKLKIVAVPKRGSAHDVLISKL
KELPKDAVIGTGSPRRMAQIYSIRSDLRGNVTTRLKKFDGLVLAEIGLKRLPPPGQGALAVIAREDNINHQSSMVSILAERTFIGKLGGGCKLP
LGAYALSLYAPENLGLTVAEEL
>ASG3_NVM04573.1_Candidatus_Helarchaeota_archaeon
IIGTRGSKLALIQAEEVISELELRIIKTHGDKFNKTPVHRKGVFVKEIDKALLEGVIDISVHSMKDLPSTIPEGIFLGAIPKRKTYKDAIYSNL
TDLKSGSIIGTGSFRRRSQVLYLRKDVRGNIDTRIKKVDAIILAKVGVQRLPAAGQGALAVETRANNINDQKASLETLAEMAFINNMGGGCQIP
IGVLGLHLKGYKKMAEKLANKI
>ASG4_NIW13145.1_Candidatus_Thorarchaeota_archaeon
TAGTRGSNLSLAQTEVVVRKLKVKVIKTTGDRITHKPLLEKGMFEKDIDKAVLRGDVDFAVHSMKDVPAIQFPKIMIVAVPERESPYDVLVSRL
KDLPNEAIVGTGSPRRKAQLHHVRPELRGNVDTRIRKLDAVIIAEAGLKRLPAPGQGALAVAAKKDNVNHWPSMAAVLAEKAFVRKIGGGCKVP
LGAIALSLYTPEELGLKVAQEI
>E1_jgi|Bigna1|90804|estExt_fgenesh1_pg.C_800006
RIGTRGSPLALAQAHETKRRLTINVISTEGDERLDIALAGKGLFTKELDVQLLLNKVDICVHSMKDVPTYLPEGTILPCNLPREDTRDVFISSL
ADLPDGSVIGSASLRRQSQILAKYPKLRGNVQTRLKKLDATMLALAGLKRMPAIAQGAIGIQCRTNDLNHPETKTCVDCERAFLAALDGNCRTP
IAGQALHFRGAVAIGKEAGEYL
>E2_jgi|Bigna1|86774|estExt_fgenesh1_pg.C_130218
RIGTRDSELAMWQAKHVESILKIVGMKTLGDKDLKSALSNKGVFTKELDAALLLGEVDIAVHSMKDLPTTLESGLSLGCVLERGTIEDAVVLSL
ATLPAGSVLGTSALRRRAVLLRNFPDLRGNLQTRLRKLDALILARTGLQRCHAVSQGALAVVCRE------------------------
GCKVPIAVTSLRLKGSLELGLQMADKV
>E3_CypaContig7377
RIGSRKSMLAMWQSEWVKARLDIMAISTK-
DKILDVPLADKGLFTKELEVELIANNVDLVVHSLKDLQTNLPDELDLGAVSDRHDPSDALVISIDDLPSGTIVGTSSLRRIAQLRHYRDDLRGN
LQTRLKKLDAIVLATAGLERLHAVGQGALGVEIRKGDINHAPTRAKCEAERAFMRELEGGCQVPIGVFTLTLTGAEALGIELANIA
>E4_YP_002049482_Paulinela
RIASRRSQLAMVQTHWVRDQLTIETMATQGDKILDVALADKGLFTKELEAQMLVNRADIAVHSLKDLPTNLPDGLVLGCVTEREDPADALVILL
DTLPNGSIVGTSSLRRLAQLRHHFPHLRGNVITRLEKLDCLILAAAGLQRLHAVGQGALGIECRDGDLEHAPTNYRCLAERAFLRELEGGCQVP
IGVNTLILTGPEDIGIKLANIL
>E5_NP_000181_Homo_sapiens
RVGTRKSQLARIQTDSVVATLEIIAMSTTGDKILDTALSEKSLFTKELEHALEKNEVDLVVHSLKDLPTVLPPGFTIGAICKRENPHDAVVFTL
ETLPEKSVVGTSSLRRAAQLQRKFPHLRGNLNTRLRKLSAIILATAGLQRMYAVGQGALGVEVRAKDLHDPETLLRCIAERAFLRHLEGGCSVP
VAVHTLYLTGAQNLGISLANLL
>E6_NP_612103_Drosophila_melanogaster
RVGSRKSELALIQTKHVIGRLEIHTMSTFGDRVLNVSLPEKSLFTRDLEDALRNGGVDFVVHSLKDLPTALPTGMAIGAVLEREDARDALVLTI
ASLPKGSVIGTSSLRRTAQIRRMYPHLRGNLNTRLAKLSGIILAQAGLVRMYAVGQGALAVECRANDLMCLNTTCRILAERSFLKTLGGGCSAP
VAVWSLSLTGANQLGKTLAEDL
>E7_XP_753473_Aspergillus_fumigatus
TIGTRKSKLALLQTDLVVAALQIHSQETAGDKNTVIALRTKNLWTQELEELLMAGDVDLIVHSLKDVPTQLPPSCTLGPMMEREDTRDVLVMSF
SEMPAGSVVGTSSIRRTAQLARRYPHLRGNIGTRLAKLTCIVLAAAGLLRLYAVGQGALGVEIRKDDIGHQKTTLACLAERSLLRTLEGGCSAP
LGVETLRMRAAEEFGVTVAKAL
>E8_XP_776442_Cryptococcus_neoformans
ILGTRKSNLALVQTGHVADDLSIESMTTVGDRNQTTPLHAKSLWTDELEARLINGHFDMLVHSLKDVPTVLKDGCEIGCMAKRHDPRDALVVRL
EDLPDGAVVGTGSVRRVAQLKRAFPNLRGNLNTRFNKLSALILAMSGLERLHAVGQGALAIEIRSTDLGHWQTEWSCGAERGCLRVLEGGCSVP
VGVESLWFSGAEQFGEECARRL
>E9_XP_001873900_Laccaria_bicolor
ILASRESQLAQIQTNSVLASFRASFMTTAGDKNQSQALYGKALWTKELEVALKEHHVDMLVHSLKDVPTTLPDGCVLGGILEREDPVDSLVVSL
DELPSGSVIGTSSVRRIAQLKRKYPHLRGNLNTRLAKLSALILAKAGLVRLYAVSQGALGIEVRSDDLTHRETQLKCLAERACLRVLEGGCSVP
VGVASLSLSGAEKVGTKLAKIL
>E11_CAMPEP_0172113374_Rhodomonas_salina
RLGTRGSPLALAQAYETRKRLQINIINTSGDMTLDKALSGKGLFTKELDVALLGNEVDFCVHSMKDVPTFLPDNTHLETMLPREDTRDAFISTV
EELPEGTIIGSASLRRQAQLLAKNPGIRGNVQTRLRKLDATLLALAGLKRMPAVAQGAIGIQVRSDDLNDKDTKICVDCERSFLATLDGSCKTP
IAGQAIHFKGFMEIGRSAGEEI
>E12_CAMPEP_0188260592_Glenodinium_foliaceum
```

RLGTRGSPLALAQAYETKRRLEIKVISTTGDERLDVPLAGKGLFTRELDVALLNKEVDFCVHSTKDVPTALVPGTELCTMLPREDTRDVIISRF
EDFPAGSVIGTASLRRQAQIYAKNPGVRGNVQTRLRKLDATLLAYAGLRRMPAIAQGAISFQCTSDDLSHQPTFDAVTCERAFLASLDGNCKTP
IAGQALRFQGAAEIGRDAGGEL
>E13_CAMPEP_0190367812_Oxyrrhis_marina
RIGTRGSPLALAQAYETRKRLEICVMKTQGDMILDKALSGKGLFTKELDVALLNNEVDICVHSMKDVPTWLPETTILPCNLPREDTRDAFICSV
QELPDGSVIGSASLRRQAQLLAKNPTLRGNVQTRLRKLDATLLALAGLKRMPAVAQGAIGIQCRENDLNHADTKTAVDCERAFLDTLDGNCKTP
IAGQALHFRG------------
>E15_KOO21739.1_Chrysochromulina_tobinii
KVGTRGSPLALAQAYETQRRLEICVIKTTGDMVLDKALKGKGLFTKELDVSLLNGAVDICVHSMKDVPTWLVDGTVLPCNLPREDTRDAFISSL
KSLKNGAVIGTASLRRQAQLLAMNPTFRGNVQTRLKKLDATLLAKAGLNRMPAVAQGAIGIQCRENDLNHQDTKVCVDCERAFLAALDGNCRTP
IAGQALEFRGAVAIGEEAGKEL
>E16_EWM30321.1_Nannochloropsis_gaditana
RIGTRGSPLALAQAYETKRRLEIKIIKTTGDMILDKALSGKGLFTKELDVALLGDEVDICVHSMKDVPTWLPDGTVLPCNLPREDSRDVFICSI
AELPAGSVIGSASLRRQAQILARNPNVRGNVQTRLRKLDATMLAVAGLNRMPAVAQGAIGIQCRSDDLNHPETKVCVDCERSFLAELDGNCKTP
IAGQALHFKGAVKIGREAGEDL
>E17_PXF48195.1_Gracilariopsis_chorda
VIGTRGSPLALAQANETKRRLEIKIIHTTGDMVLDKALAGKGLFTKEIDIAQLKGEIDIAVHSMKDVPTWLPDGIILPCMLPREDTRDVFISCI
EDLPDGSVIGSASLRRQSQILAKNPTLRGNVQTRLRKLDATLLALAGLSRMPAVAQGAIGITCREDDLNHKETKTCVGCERSFLAKLDGSCRTP
IAGQALQFRGAIAIGTDAGIEL
>E18_CBJ30046.1_Ectocarpus_siliculosus
TIGTRGSPLALAQAYETRKRLAIQVIKTSGDMILDKPLTGKGLFTKELDVQLLNKDVDICVHSMKDVPTWIVPGTILPCNLPREDTSDAFISNI
ASLPDGSVIGSASLRRQAQLLRANPTFRGNVQTRLRKLDATLLAFAGLKRLPAVAQGAIGIQCRENDLCHQETKTAVDCERAFLAALDGNCKTP
IAGQALVFRGAVAMGHDAGMEL
>E19_XP_009037531.1_Aureococcus_anophagefferens
RIGTRGSPLALAQAYMTRELLQLCIMKTQGDMILDKALSGKGLFTKELDVALLGDEVDICVHSMKDVPTWLPAKTVLPCMLEREDTRDVFISSL
KDLKDGSVIGSASLRRQSQILAINPTLRGNVQTRLRKLDATLLAYAGLKRMPAVAQGAIGIQCRDDDLNHEETFTCVSCERAFLAALDGNCKTP
IAGQALLFDGAEKIGREAGEGI
>E22_VEU41363.1_Pseudo_nitzschia_multistriata
RIGTRGSPLAMAQAFETRRRLEICVMKTQGDMILDKSLMGKGLFTKELDVALLGDEVDICVHSMKDVPTWLPEGTVLPCNFPREDTNDAFITSI
ADLPDGSVIGTASLRRQAQILSQNPTLRGNVQTRLRKLDATLLAIAGLKRMPAVAQGAIGVQCREGDLNDMDTHVCVNCERAFLEALDGNCKTP
IAGQAIKFRGAVKIGKEAGEKL
>E23_OEU23734.1_Fragilariopsis_cylindrus
RIGTRGSPLAMAQAFETRRRLEICVMKTQGDMILDKSLMGKGLFTKELDVALLANEVDICVHSMKDVPTWLPEGTVLPCNLPREDTNDAFITTI
ADLPDGSVIGTASLRRQSQLMAQNPTLRGNVQTRLRKLDATLLAIAGLKRMPAVAQGAIGIQCRTDDLNHMDTHVCVNCERAFLEALDGNCKTP
IAGQAIKFRGAVKIGHEAGEKL
>E25_XP_002292986.1_Thalassiosira_pseudonana
RIGTRGSPLALAQAYETRRRLEICVMKTQGDMILDKSLMGKGLFTKELDTALLGDEVDICVHSMKDVPTWLPEGTVLPCNLPREDTNDAFITRI
ADLPDNSVIGTASLRRQAQILAQNPTLRGNVQTRLRKLDATLLAIAGLKRMPAVAQGAIGIQCRSDDLNCMDTHVCVNCERAFLEALDGNCKTP
IAGQAIVFKGAVEIGRKAGEEL
>E26_XP_002179459.1_Phaeodactylum_tricornutum
RIGTRGSPLALAQAYETRKRLEICVLKTQGDMILDKSLMGKGLFTKELDTALLSDEVDICVHSMKDVPTWLPDGTVLPCNLPREDTNDAFIYRI
EDIPDGSVIGTASLRRQAQLMAKNPTLRGNVQTRLRKLDATLLAIAGLKRMPAVAQGAIGIQCRDDDLNHPETKACVDCERGFLEALDGNCKTP
IAGQAIMFRGAAKIGREAGEGL
>E27_ABD51930.1_Guillardia_theta
KLGTRGSPLALAQAYETRRRLSINIINTSGDMELSKALSGKGLFTKELDVALLKKEVDFCVHSMKDVPTYLPDGTHLEAMLPREDTRDAFISSF
EEMPEGTVIGSASLRRQAQIFAKNPKIRGNVQTRLRKLDATLLALAGLKRMPAVAQGAIGIQVRSDDLNHKDTKTCVDCERSFLATLDGSCKTP
IAGQAIHFRGYMKIGKDAGEEI
>E27_OSX71464.1_Porphyra_umbilicalis
VIGTRGSPLALAQAHETQRRLEIRVIHTTGDMVLDKALSGKGLFTKEIDVAQLNGDVDIAVHSMKDVPTWLPDGIVLPCMLPREDTRDVFISCL
ADLPDGSVIGSASLRRQSQILAKFPTLRGNVQTRLKKLDATMLALAGLARMPAVAQGAIGITCRAGDLTHHETLDCVRAERAFLEALDGSCRTP
IAGQAIAFRGGVAMGKDAGEEL
>E28_KAA8492666.1_Porphyridium_purpureum
VIGTRGSPLALAQAYETRRRLDISVINTSGDMFLDTTLAGKGLFTKEIDSAQLRGDVDIAVHSMKDVPTWLPNGIELPCMLPREDTRDAFISGL
SDLPDGSVIGSASLRRKSQLLAMNPTLRGNVQTRLRKLDATLLAIAGLNRMPAVAQGAIGITCRSNDLNHADTLTCVSCERSFLAALDGSCRTP
IAGQALKFRGAVKIGADAGAEL
>E31_XP_024378864.1_Physcomitrium_patens
RIGTRGSPLALAQAYQTRDKLEIVIIKTTGDKILSQPLAGKGLFTKEIDDALLNGDIDIAVHSMKDVPTYLPEGTILPCNLPREDVRDAFICSL
AELPEGSVVGSASLRRQSQLLHKYPHLRGNVQTRLRKLQATLLALAGLKRLPAIAQGAIGIACRTGDLNHEDTRLAVACERAFLAKLDGSCRTP
IAGLACSFRGMIALAQDAGQEL
>E32_OAE31707.1_Marchantia_polymorpha
RIGTRGSPLALAQAYQTRDKLEIVIIKTTGDKILNQPLAGKGLFTKEIDDALLGGSIDIAVHSMKDVPTYLPEGTILPCNLPREDVRDAFICSL
AELPAGSVVGTASLRRQSQLLNKYPSLRGNVQTRLRKLQATLLALAGLKRLPAIAQGAIGIACRSDDLNHEDTRLAISCERSFLAALDGSCRTP
IAGLACTFRGMIAVGKDAGEEL
>E33_CEM32015.1_Vitrella_brassicaformis
IIGTRGSPLALAQAHETKRRLELKIIKTTGDERLDIALAGKGLFTKELDVALLAKEVDICVHSMKDVPTYLPEGTVLACNLPREDTRDVFISTI
RELPDGSVVGSASLRRQAQILAINPTLRGNVQTRLKKLDATMLALAGLKRMPAVAQGAIGIQCRSDDLNCPDTKRCVDCERAFLAQLDGNCRTP
IAGQALKFRGAVNIGKQAGEEL
>E34_XP_003607331.1_Medicago_truncatula
RIGTRGSPLALAQAHETRDKLQIVIIKTTGDKILSQPLAGKGLFTKEIDEALINGDIDIAVHSMKDVPTYLPEKTILPCNLPREDVRDAFISSL
ADLPAGSVIGTASLRRKSQILHRYPSLRGNVQTRLRKLKATLLALAGLKRLPAVAQGAIGIACRSNDLNHEETRLAISCERAFLTTLDGSCRTP
IAGYACLFRGMMKMGRDAGEEL
>E35_XP_002507564.1_Micromonas_commoda
KIGTRGSPLALAQAYMTRDLLEICIIKTTGDKVLDQPLAGKGLFTRELDVALLDGRIDIAVHSMKDVPTYLPEGTILPCMLPREDVRDAFISDL
SELPEGSLVGTASLRRQSQLLARFPGLRGNVQSRIRKLDCTLLAIAGLNRMPAVAQGAIGIACRTGDLNHEETRIAVECERSFLAALDGSCRTP
IAGFALRFNGAIAAGKEAGAEL
>E36_XP_025653893.1_Arachis_hypogaea

RIGTRGSPLALAQAYETRDKLQIVIIKTTGDKILSQPLAGKGLFTKEIDEALLNSEVDIAVHSMKDVPTYLPDGTILPCNLEREDVRDAFISSL
ADLPSGSVVGTASLRRKSQILHRYPSLRGNVQTRLRKLQATLLALAGLKRLPAVAQGAIGIACRSNDLNHEETRLAVVCERAFLTTLDGSCRTP
IAGYACLFRGMIEMGKDAGKEL
>E37_XP_006648352.2_Oryza_brachyantha
RIGTRGSPLALAQAHETRDKLEIIIIKTTGDMILDKPLAGKGLFTKEIDDALLQGRIDIAVHSMKDVPTYLPEGTILPCNLPREDVRDAFICSL
AELPAGSVVGSASLRRQSQILYRYPSLRGNVQTRLRKLHATLLALAGLKRLPAVAQGAIGIACRSNDLNHEDTRLAVACEREFLSVLDGNCRTP
IAAYACSFRGMVEMGKDAGHEL
>E39_XP_003570067.1_Brachypodium_distachyon
RIGTRGSPLALAQAHETREKLEIIIIKTTGDMILDKPLAGKGLFTKEIDDALLQGRIDIAVHSMKDVPTYLPEGTILPCNLPREDVRDAFICTL
AELPAGSVVGSASLRRQSQILYKYPSLRGNVQTRLRKLHATLLALAGLKRLPAVAQGAIGIACRTNDLNHEDTRLAVACEREFLSVLDGNCRTP
IAAYACSFRGMVALGQDAGYEL
>E40_CBI18797.3_Vitis_vinifera
RIGTRGSPLALAQAYETRDKLQIVVIKTTGDKILTQPLAGKGLFTKEIDEALLNGDIDIAVHSMKDVPTYLPDKTILPCNLPREDVRDAFISSL
SELPAGSIVGTASLRRKSQLLHRYKSLRGNVQTRLRKLQATLLALAGLKRLPAVAQGAIGIACRSNDLNHEVTRLAVACERAFLETLDGSCRTP
IAGYACIFKGMIKMGKDAGEEL
>E41_GAX77900.1_Chlamydomonas_eustigma
KIGTRGSPLALAQAYMTRDLLEITIIKTTGDKILNQPLAGKGLFTKEIDDALLDGRVDIAVHSMKDVPTYLPAGTILPCNLPREDVRDVFISSL
AELPAGAVVGSASLRRQAQILNKYPHLRGNVQTRLRKLSATLLALAGLKRLPAVSQGAIGIACRDGDLNHEETRIAIVCERAFLTALDGSCRTP
IAGYALHFRGAITVGTECGAEL
>E48_XP_005782931.1Emiliania_huxleyi
KLGTRGSPLALAQAYETKRRLEIKVIKTTGDMVLDKALKGKGLFTKELDVSLLNGDVDCCVHSMKDVPTWLVPGTILPCNLPREDTRDAWISLP
QELPDGAVIGTASLRRQAQLYAINPTFRGNVQTRLKKLDATMLAKAGLNRMPAVAQGAIGIQIREKDLNHEPTKVAVDCERAFLAALDGNCRTP
IAGQALRFEGAAKMGTEAGEEL
>E49_KAF4358603.1_Cannabis_sativa
RIGTRGSPLALAQAYETRDKLEIVIIKTTGDKILTQPLAGKGLFTKEIDEALINSDIDIAVHSMKDVPTYLPEKTILPCNLPREDVRDAFISSL
AELPEGSVIGTASLRRKSQILHRFPSLRGNVQTRLRKLQATLLALAGLKRLPAVAQGAIGIACRSDDLNHEETRLAVACERAFLLTLDGSCRTP
IAGYACIFRGMVSMGKDAGKEL
>C1_WP_008276417.1_Crocosphaera_chwakensis
RIGSRKSQLALVQTYWIQEQLDVETMSTQGDKILDVALADKGLFTKELETAMLQNQVDFAVHSLKDLPTNLPDGLMLGCVTERVNPADALVVQL
DTLPEGSVIGTSSLRRLAQLRHHYPHLRGNVNTRLSKLDAIILAVAGLQRLHAVGQGALGIECRTGDLEHPETRDRCYAERAFLRELEGGCQVP
IGVNTLTLTGAEKLGQELAMRL
>C3_HAC63707.1_Cyanothece
RIGSRKSQLALVQTYWVQKELDVETMSTQGDKILDVALADKGLFTKELEVAMEQNQIDLAVHSLKDLPTNLPKGLMLGCITERVDPADALVIQL
ETLPEGAVIGTSSLRRLAQLRHHFPHLRGNVNTRLRKLDAIILAVAGLQRLHAVGQGALGIECREGDLEHEPTRDRCYAERAFLRELEGGCQVP
IGVNTLTLTGAEQLGLELANLL
>C4_WP_046663363.1_Microcystis_aeruginosa
RIGSRKSQLALVQTYWVQAELEVETMSTQGDKILDVALADKGLFTKELEVGMLDRSIDLAVHSLKDLPTNLPEGLILGCVTERVNPADALVVQL
DTLPAGAVVGTSSLRRLAQLRYHYPHLRGNVNTRLAKLDAIILAVAGLERLHAVGQGALGIECRDGDLEHAPSRDRCYAERAFLRELQGGCQVP
IGVNTLTLTGGEVLGRDLAHKV
>C5_WP_107666849.1_Cyanothece
RIGSRKSQLALVQTYWIQEQLDVETMSTQGDKILDVALADKGLFTKELETAMLQNQVDFAVHSLKDLPTNLPEGLMLGCVTERVNPADALVVQL
DTLPEGSVIGTSSLRRLAQLRHHYPHLRGNVNTRLSKLDAIILAVAGLQRLHAVGQGALGIECRSGDLEHPETRDRCYAERAFLRELEGGCQVP
IGVNTLTLTGAEKLGQELALRL
>C7_ACB53726.1_Crocosphaera subtropica ATCC 51142]
RIGSRKSQLALVQTYWIQEELDVETMSTQGDKILDVALADKGLFTKELETAMLQNQVDFAVHSLKDLPTNLPEGLMLGCVTERVNPADALVVQL
DTLPEGSVIGTSSLRRLAQLRHHYPHLRGNVNTRLSKLDAIILAVAGLQRLHAVGQGALGIECRSGDLEHTETRDRCYAERAFLRQLEGGCQVP
IGVNTLTLTGAEKLGEELALRL
>C9_WP_152589085.1_Nostoc_sphaeroides
RIGSRKSQLALVQTYWVQEQLEVHTMSTQGDKILDVALADKGLFTKELEVGMLNQEIDFAVHSLKDLPTHLPEGLTLAAITERENPADALVVQI
DTLPEGAVIGTSSLRRLAQLRHHFPHFRGNLITRLAKLDALILAAAGLERLHAVGQGALGIECRADDIEHPETRDRCLAERSFLRSLEGGCQVP
IGVNTLTLTGAEALGIELAQVL
>C10_WP_159290979.1_Microcystis_aeruginosa_
RIGSRKSQLALVQTYWVQAELEVETMSTQGDKILDVALADKGLFTKELEVGMLDRSIDLAVHSLKDLPTNLPEGLILGCVTERVNPADALVVQL
DTLPEGAVVGTSSLRRLAQLRYHYPHLRGNVNTRLAKLDAIILAVAGLERLHAVGQGALGIECRDGDLEHAPSRDRCYAERAFLRELQGGCQVP
IGVNTLTLTGGEVLGRDLAHKV
>C11_WP_073548994.1_Chroogloeocystis_siderophila
RIGSRKSQLALVQTYWVQEQLEVHTMSTQGDKILDVALADKGLFTKELEVGMQSHEIDFAVHSLKDLPTNLPEGLMLGVVTEREDPADALVVQI
DTLPEGAVIGTSSLRRLAQLRHHFPHLRGNLNTRLAKLDAIILAVAGLRRLHAVGQGALGIECRTDDIEHVPTRDRTLAERAFLRSLEGGCQVP
IGVNTLTLTGAEKLGTELAQRL
>C12_WP_039716212.1_Scytonema_millei
RIGSRKSQLALVQTYWVQEQLEVHTMSTQGDKILDVALADKGLFTKELEVGMLNQEIDFAVHSLKDLPTRLPQGLMLGAVTERENPADALVLQF
DTLPEGAVIGTSSLRRLAQLRYHFPHLRGNVITRLQKLDALVLAVAGLQRLHAVGQGALGIECREGDIEDVPTRDRALAERAFLRELEGGCQVP
IGVNTLTLTGAEKLGIELAQRL
>AP2_NVK18543.1_Methylocystaceae_bacterium
VIGTRGSPLALAQAHETRDRLEIEVIKTTGDMILDRPLSGKGLFTKEIDDAQLNGRIDIAVHSMKDVPTWLPDGISLPCILPREDVRDVFISTL
ADLPKGAVIGSASLRRQAQIKAAYPHLRGNVQSRLRKLDATLLANAGMRRLPAVAQGAIGISCRSNDLNCATSHIRILAERALLRVLDGSCRTP
IAALAIRLRGPDGLGTKAGLEL
>AP4_GGF59690.1_Terasakiella_brassicae
VIGTRGSPLALAQAHETRDRLEIEVIKTTGDMILDRPLSGKGLFTKEIDNAQLEGRIDIAVHSMKDVPTWLPEGIDLPCILPREDVRDVFISCL
ADLPAGSVVGSASLRRQAQIKALYPHLRGNVQRRLSKLDATLLAHAGLRRLPAVAQGAIGISCRSNDLHCAQSATRIEAERALLRILDGSCRTP
IAALAIVLRGPDGLGTKAGQEL
>AP5_WP_135081589.1_Terasakiella_sp
VIGTRGSPLALAQAYETRERLEIEVIKTTGDMILDRPLSGKGLFTKEIDDAQLDGRVDIAVHSMKDVPTWLPDGLCLPCILPREDVRDVFISTL
ADLPQGAVVGTASLRRQAQIKAKYPHLRGNVQSRLRKLDATLLANAGLRRLPAVAQGAIGITCRENDLNCETSKIRIEAERALLRVLDGSCRTP
IAALAITLRGPDSLGTRAGEEL
>AP7_WP_011391315.1_Rhodospirillum_rubrum

RIGTRGSPLALAQTHQVRDLLTIEVIKTTGDAILDRPLSGKGLFTREIDDAMLAGTIDIAVHSMKDVPTYLPDGIVLPCMLEREDVRDAFLGRL
ADLPEGSVVGTASLRRGAQILAMRPDIRGNVQTRLDKLDATMLAIAGLNRLPAVAQGAVGVTCRAGDLNHRATQLCVEIERAFLTRLDGSCRTP
IGGLAVFFRGGIALGDDAGAEL
>AP8_GEO82801.1_Pararhodospirillum_oryzae
MIGTRGSPLALAQTHQVRDLLGIEIIKTTGDAILDRPLAGKGLFTKEIDEAMLDGRIDIAVHSMKDVPTYLPPGIVLPCVLEREDVRDAFLGTL
ESLPTGAVVGTSSLRRGAQILAMRPDLRGNVQTRLDKLDATMLAKAGLNRLPAVAQGAVGCTCRADDLHHAETSLCVDVERAFLARLDGSCRTP
IGGLAVFFRGAVALGEDAGAEL
>AP9_WP_068493424.1_Magnetospirillum_marisnigri
RIGTRGSPLALAQTHETRDRLEIEVIKTTGDLVQDRPLAGKGLFTKELDEAMLSGRIHLAVHSMKDVPTLLPDGIDLPCILPREDVRDAFISSL
ADLPEGAVIGSASLRRAAQILNRRPDLRGNVQTRLRKLDATLLAMAGLRRLPAVAQGAIGITCRSDDLNCLDSQVRVSAERAFLARLDGSCRTP
IAALALSFRGAEALGRDAAEEL
>AP12_WP_024079153.1_Magnetospirillum_gryphiswaldense
TIGTRGSPLALAQTHETRDRLAIQVIQTTGDLIQNRPLAGKGLFTKELDDSMLDGRIDLAVHSMKDVPTVLPDGIVLPCVLPREDVRDAFLSGI
DDLPQGAVVGTASLRRGAQILHRRPDLRGNVQSRLRKLDATLLAMAGLNRLPAVAQGAIGITCRADDLNCPETMIRITAERAFLLTLDGSCRTP
IAALALSFRGAHAMGVDAGREL
>AP16_WP_114121889.1_Thalassospira_xianhensis
RIGTRGSPLAMAQAHEVRDKLAITVITTTGDKILDRALSGKGLFTKEIDDALMGGEIDLAVHSMKDVPTVLPDGMILPTILPREDVRDAFISSF
AEMPAGSVIGSASLRRQAMILNKYPDLRGNVQTRLRKLDATLLAMAGLNRLPAVAQGAIGITIRENDLNCPASAICVTAERAALRALDGSCRTP
IAALAMRLRVAATAGTDAGNEL
>AP18_WP_022732749.1_Thalassospira_lucentensis
RIGTRGSPLALAQAHEVRDKLAITVITTTGDKILDRALSGKGLFTKEIDDALMGGDIDLAVHSMKDVPTVLPDGMILPTILPREDVRDAFISSF
AEMPAGSVIGSASLRRQAMILNKYPDLRGNVQTRLRKLDATLLAMAGLNRLPAVAQGAIGITIREDDLHCEKSAIRVAGERAALFALDGSCRTP
IAALAMRLRVASAAGKDAGEEL
>AP20_MBB21022.1_Rickettsiales_bacterium
RIATRKSALAMWQAEYVQAKLELVPMSTQGDRILDTPLAGKGLFIKELEVAMSEGRADIAVHSMKDVPVDFPEGFGLHCICERENPYDAFVSSI
EELPQGAIVGTSSLRRQCQIRSARPDLRGNVNTRLAKLDAIILAAAGLIRLPAVGQGAVGIECRNDDLNHSNTESRVKAERAMNAKLNGGCQVP
IGSYALYLRGAISIGVDVAEQL
>AP21_WP_143689836.1_Wolbachia_Nilaparvata_lugens
KIGTRGSDLAIIQALEAKQRLEIIKIKTSGDKYANVTLAGKGLFLREIEAELLENNIDMAVHSLKDVPAFFSKDLTIPCVLERLSPCDVFVSSL
KTLPQQATIATSSIRRKVQLLNFRPDLRGNVTTRLQNQDGMILAKAGLIRLNAVGQGAICIQCRKNDINNNMSFIRAKSERSFMKTVNGSCFTP
LAALALRLRCAEKIGMDAGLEL
>AP23_WP_167896255.1_Wolbachia_Brugia_pahangi
RIGTRGSSLAIAQALEAKQKLEIIKIKTSGDKYANANLAGKGLFIKEIETELIKSNIDMAVHSLKDVPAFFSKDLTIPCILERLSPYDTFISSL
KALPQQAIIATSSIRRKVQLLNFRPDLRGNVTTRLQNQDGIILAEAGLIRLSAVGQGAICIQCRKNDINNNKSFIRVKSERSFMKTVNGSCFTP
LAALALHLYCAEKMGMDAGLEL
>AP24_WP_182367144.1_Wolbachia_pipientis
KIGTRGSKLAVAQALEAKQKLEIVKIKTSGDKYANANLAGKGLFIKEIETELLENNIDMAVHSLKDMPAFFSGGLTIPCVLERLSSCDAFISSL
ESLPQQATIATSSIRRKVQLLNVKPDLRGNVTTRLQNQDGIILAEAGLIRLSAVGQGAVCIQCRRNDINNNMSFIGVKSERSFMKTVNGSCFTP
LAALALHLRCAEKMGMDAGLEL
>AP25_QLK56876.1_Ehrlichia_ruminantium
YVGTRGSILAIAQAMEVKKLLQIVHIVTSGDINDKISLSGKGLFLKELEEALLTGTIDLAVHSMKDVPAFYCDGLVIPCILKRISPYDVFISSL
KSLPDNAVIGTSSIRRKVQLMRLLSSVRGNVDTRILKLDGIILAKAGLMRISAVGQGAIGIQCRANDLNCKKSYISVAAERSFMKTVNGSCDTP
LAALALYMSCAEISGINMGNNL
>AP26_WP_011114401.1_Anaplasma_marginale]
RLGTRGSTLALIQAEEVKAAIDVTKIKTSGDIKVDRPLCGKGLFIKEIEEALLANKIDIAVHSAKDVPGFYSENLDMPCVLKRRSPMDVFISSL
NSLPAGAKIGTSSIRRKVQLLAFRPDLRGNVDTRIAKVDGIVLAEAGVDRIGAVGQGAICVQCRKSDLNDHESFTAVLAERSFMKAVNGSCHTP
LAAMAMSMKCAESVGFEMGVML
>AP27_WP_040255795.1_Rickettsia_hoogstraalii
RIGTRKSPLALTQTNLVIQQIEIVPIITSGDLIQNKPLYGKALFLKEIEQALLDKKIDLAVHSLKDVPGRIPEELVIAAVLEREDPRDVFVCSI
EELPQNAVIGSSAVRRKAFIQKIRPDLRGNVDSRIKKLDATILAYAGLKRLPCIGQGVIAVEIRQNDINHIPTFELIKPERAFLEYLDANCRTP
IAAYSIKNNFSKEAGIKAAKMM
>AP28_WP_012148629.1_Rickettsia_canadensis
RIGTRKSPLALIQTNLVIQQIKIVPITTSGDLIQNKPLYGKALFLKEIEQALLDKKIDLAVHSLKDVPGRIPEDLIIAAVLEREDPRDVFVCSI
EELPQNAIIGSSAVRRKTFIEKIRSDLRGNVDSRIKKLDATILAYAGLKRLPCIGQGVIAVEIRKDDINHLTTFELIKPERAFLEYLDANCRTP
IAAYSIETDFSKEAGIKAAKIM
>AP29_WP_011477036.1_Rickettsia_bellii
RIGTRKSKLALTQTNLVIEQIEIVEITTSGDLIQNKPLYGKALFLKEIEQALLNKKVDLAVHSLKDVPGIIPKDLSIEAVLEREDSRDVFVCSI
EELPKNAVIGTSSVRRKLCVQRMRPDLRGNVDSRINKLDATILAYAGLKRLPCIGQGVIAIEIRKDDINHLPTFELIKPERALLEYLDANCRTP
IGAYSIQTDFSKESGIKAAKVM
>AP31_KJV80341.1_Rickettsia_hoogstraalii
RIGTRKSPLALIQTNLVIQQIEIVPIITSGDLIQNKPLYGKALFLKEIEQALLDKKIDLAVHSLKDVPGRIPEELVIAAVLECEDPRDVFVCSI
EELPQNAVIGSSAVRRKAFIQKIRPDLRGNVDSRIKKLDATILAYAGLKRLPCIGQGVIAVEIRQNDINHIPTFELIKPERAFLEYLDANCRTP
IAAYSIKTNFSKEAGIKAAKMM
>AP32_WP_032138717.1_Rickettsia_tamurae
RIGTRKSPLALIQTNLVIQQIEIVPIITSGDLIQNKPLYGKALFLKEIEQALLDKKIDLAVHSLKDVPGRIPEELVIAAVLEREDPRDVFVCSI
KELPQNAVIGSSAVRRKAFIQKIRPDLRGNVDSRIKKLDATILAYAGLKRLPCIGQGVIAVEIRQDDINHIPTFELIKPERAFLEYLDANCRTP
IAAYSIQTDFSKEAGIKAAKMM
>AP33_WP_041079570.1_Rickettsia_asembonensis
RIGTRKSPLALIQTNLVIQQIEIVPIITSGDLIQNKPLYGKALFLKEIEQALLDKKIDLAVHSLKDVPGRIPEELVIAAVLEREDPRDVFVCSI
EELPQNAVIGSSAVRRKAFIQKIRPDLRGNVDSRIKKLDATILAYAGLKRLPCIGQGVIAVEIRQNDINHIPTFELIKPERAFLEYLDANCRTP
IAAYSIQTDFLKESVIKAAKMM
>AP34_WP_015508436.1_Rickettsia_prowazekii
RIGTRNSTLALIQTNLVIEQIEIVPIITSGDLIQNKPLYGKALFLKEIEQALLDKKIDLAVHSLKDIPGKIPVELVIAAVLEREDPRDVLVCSI
ETLPQNAVIGSSAVRRKAFIKKLRPDLRGNVDSRIKKLDAIILSYAGLKRLPCIGQGVIAVEIRKDDINHLPTFELIKPERAFLEYLDANCSTP
IAAYAIQIDFSKICGIKAAKMM
>AP42_WP_103897011.1_Rickettsia_fournieri

RIGTRKSPLALIHTNLVIQQIEIVPIITSGDLIQNKPLYGKALFLKEIEQALLDKKIDLAVHSLKDVPGRIPAPLVIAAVLEREDPRDVFVCSI
EELPQNAVIGSSAVRRKAFIQKIRPDLRGNVDSRIKKLDATILAYTGLKRLPCIGQGVIAVEIRKDDINHLPTFELIKPERAFLEYLDANCRTP
IAAYSIQTNFSTEAGIKAAKMM
>AP43_WP_045800397.1_Rickettsia_amblyommatis
RIGTRKSPLALIHTNLVIQQIEIVPIITSGDLIQNKPLYGKALFLKEIEQALLDQKIDLAVHSLKDVPGRIPEPLVIAAVLEREDPRDVFVCSI
EELPQNAVIGSSAVRRKAFIQKIRPDLRGNVDSRMKKLDATILAYTGLKRLPCIGQGVIAVEIRKDDINHLPTFELIKPERAFLEYLDANCRTP
IAAYSIQTDFSTEAGIKAAKMM
>AP44_WP_010422890.1_Rickettsia_helvetica
KIGTRKSPLALIQTNLVIQQIEIVSIITSGDLIQNKPLYGKALFLKEIEQALLDKKIDLAVHSLKDIPGRIPEELVIAAVLEREDPRDVFVCSI
EELPQNAVIGSSAVRRKAFIQKIRMDLRGNVDSRIKKLDATILAYAGLKRLPCIGQGVIAVEIRQDDINHLPTFELIKPERAFLEYLDANCCTP
IAAYSIKTDFSKEASIKAAKMM
>AP45_WP_145622287.1_Nitrospirillum_amazonense
RIGTRGSPLALAQAHETRDRLEIVVIKTTGDRILDRTLAGKGLFTKEIEDALLAGEIDLAVHSMKDVPTVLPEGLDITCLLPREDPRDAWFSTL
MELPAGAVVGTASLRRQAQVLARRPDLRGNVQTRLRKLDATLLAMAGLRRLPAVAQGAIGIEARIADLNCADTALRVTAERGVLTALDGSCRTP
IAALAMDMEAARAAGLDAGAEL
>AP46_WP_044558528.1_Azospirillum_sp
RIGTRGSPLALAQAHETRDRLEIVVIKTTGDRILDRTLAGKGLFTKEIEDALLAGEVDLAVHSMKDVPTVLPDGLGITCLLPREDPRDAWFSGL
LDLPAGAVVGTASLRRQAQVLARRPDLRGNVQTRLRKLDATLLAMAGLRRLPAVAQGAIGIETRLDDLNCAETALRVTAERGVLTALDGSCRTP
IAALAMDLEAALAAGLDAGQEL
>AP48_WP_014415752.1_Pararhodospirillum_photometricum
TIGTRGSPLALAQTHQVRDLLAIEVIKTTGDAILDRPLAGKGLFTKEIDEAMLSGRIDIAVHSMKDVPTYLPDGIVLPCMLEREDVRDAVIGSI
DALPLGAVVGTASLRRGAQILAKRPDLRGNVQTRLAKLDATLLAKAGLNRLPAVAQGAVGVTCRADDLNHAETFLCVTVERAFLAKLDGSCRTP
IGGLAVFFRGAVALGDDAGAEL
>AP51_WP_008616862.1_Magnetospirillum_caucaseum
RIGTRGSPLALAQTHETRDRLDIEVIKTTGDLVQDRPLAGKGLFTKELDEAMLSGRIHLAVHSMKDVPTQLPDGIVLPCILAREDVRDAFISSL
ADLPQGAVVGTSSLRRGSQILHRRPDLRGNVQTRLRKLDATLLAMAGLRRLPAVAQGAIGITCRAGDLNCPDSFVRVAAERAFLTRLDGSCRTP
IAALALSFRGAEAMGKDAAEEL
>AP55_WP_074770425.1_Phaeospirillum_fulvum
RIGTRGSPLALAQTYETRERLEIEVIKTTGDMVLDRPLSGKGLFTRELDEAMLDGRIRIAVHSMKDVPTYLPDGIVLPCILPREDVRDAFICSI
ADLPAGSVIGSSSLRRGAQILNRRPDLRGNVQTRMRKLDATLLAMAGMRRLPAVAQGAIGITCRADDLNCKDSFDRVAAERAFLTKLDGSCRTP
IAALAMLFRGGVAMAIDAAEEL
>AP57_WP_043745666.1_Magnetospirillum_magneticum
RIGTRGSPLALAQTHETRDRLDIEVIKTTGDLVQDRPLAGKGLFTKELDEAMLSGRIHLAVHSMKDVPTQLPDGIVLPCILPREDVRDAFLSSL
AELPQGAVVGTSSLRRGAQILHRRPDLRGNVQTRLRKLDATMLAMAGLRRLPAVAQGAIGITCRADDLNCPDSFVRVAAERAFLTRLDGSCRTP
IAALALSFRGAEAMGKDAAEEL
>AP59_WP_042695698.1_Azospirillum_sp
RIGTRGSPLALAQAHETRDRLEIVVFKTTGDRILDRTLAGKGLFTKELEEALFDGRADLAVHSMKDVPTQLPDGLEIATLLPREDPRDAFFSGL
ADLPAGAVVGTAGLRRQAQVLELRPDLRGNVQTRLSKLDATLLALAGLRRLPAVAQGAIGIEIRSDDLNCAETMVRVTAERALLAALDGSCRTP
IAALALHLRAAESMGADAGAEI
>AP60_WP_155189669.1_Roseibium_denhamense
RIGTRGSALALAQAEETRDRLEIVVIKTSGDRIQDRPLSGKGLFTKEIEEALLDKSIDIAVHSSKDMPTVLPDGLELTAFLPREDVRDAFLSTL
TDLPHGAVVGSSSLRRQAMIRRLRPDIRGNLQTRMRKLDATLLAAAGLKRLPAVGQGAICIESRSGDIHDPETQIRLDAERAFLAVLDGSCRTP
IGGLALRFKGAVAIGEAIGQEL
>AP61_WP_007255416.1_Oceanicola_granulosus
KIGTRGSPLALAQAHETRDRLEVVVIRTTGDRVVDRPLKGKGLFTKEIEEAMLSGAIDIAVHSMKDMPVAQPEGLVLDCYLPREDPRDAFVSGI
KALPDGATVGTSSLRRKAQLLHRRPDLRGNVQTRLRKLVATFLAVAGLNRLPAVAQGAIGIERRASDIHDRATGERLAAERAFLAALDGSCETP
IAGLALRLRGGAALGTDMARRL
>AP62_PID35544.1_Rhodobacterales_bacterium
RIGTRGSPLALAQAYETRRRLEIVVIKTTGDDRADRPLKNKGLFTKEIEEQLLSGEIDIAVHSSKDMPTEQPEGLRLDCFLPREDPTDAFVSSL
AELPEGAVVGTSSLRRRAQVLHRRPDLRGNVQTRMRKLDATFLAMAGINRLPAVAQGAIGIERRVGDIQDAETGQRLAAERAFLAALDGSCETP
IAGLALHLRGAESLGAALAQDL
>AP63_WP_085869129.1_Pseudoruegeria_aquimaris
KIGTRGSPLALAQARETRARLEIVVIKTTGDDRADRPLKNKGLFTKEIEEAMLAGTIDIAVHSSKDMPVEQPQGLVLDCFLPREDVRDAFISNI
AALPPGAVVGTSSLRRKAQLLNVRPDLRGNVQTRLRKLQCTFLAMAGLNRLPAVAQGAIGIERRESDLHCTETGTRLAAERAFLARLDGSCETP
IAGLALTLRGAKALGTELAETL
>AP64_WP_148633458.1_Rhodobacterales_bacterium
RIGTRGSALALAQAHETRDRLEIVVIKTSGDRIQDRPLSGKGLFTKEIEEALLDGRIDLAVHSSKDMPTVLPDGLALTAFLPREDVRDAFLSTL
EDLPHGAVVGSSSLRRQAMIKRLRPDIRGNLQTRLRKLDATLLAAAGLRRLPAVGQGAICIESRENDIHDQETQIRLDAERAFLAVLDGSCRTP
IGGLALHFKGAVEIGKAAGAVL
>AP65_WP_121163363.1_Roseovarius_spongiae
KLGTRGSPLALAQAYETRARLEIVVIKTTGDRVIDRPLKGKGLFTREIEQALLAGRIDLAVHSMKDMPVLQPEGLLLDTYLPREDPRDAFVSGL
AELPDGAKVGTSSLRRKAQVLVAYPALRGNVQTRLKKLACTFLAVAGLNRLPAVAQGAIGIERRADDIHDHETGLRIAAERAFLAELDGSCETP
IAGLALRLRGGAELGRAMARDL
>AP66_WP_106753742.1_Pannonibacter_carbonis
RIGTRGSALALAQAHETRSRLDIVVIKTSGDQIQDRPLSGKGLFTKEIEEALLDGRIDIAVHSSKDMPTHLPGGLALTAFLPREDVRDAFISRL
EDLPQGAVVGSSSLRRQAMIKRLRPDIRGNVQTRLKKLDATLLAYAGLRRLPAVGQGAICIESRAADIHHRETELRLTCERAFLAELDGSCRTP
IGGLALSFRGAEAIGREAGRSL
>AP68_WP_009806314.1_Pseudooceanicola_batsensis
KIGTRGSPLALAQAHEARDRLEVCVIQTTGDRVQDRPLKGKGLFTREIEHALLTGGIDIAVHSMKDMPVLQPEGLLLDCYLPREDPRDAFVSRL
DQIPAGARVGTSSLRRRAQVLVRRPDLRGNVQTRLRKLSATFLAMAGLNRLPAVAQGAIGIERREDDIHDPETARQLAAERAFLAALDGSCETP
IAGLALRLRGGAKLGREMGAAL
>AP69_WP_075283376.1_Labrenzia_aggregata
RIGTRGSALALAQAHETRDRLEIVVIKTSGDRIQDRPLSGKGLFTKEIEEALLDGRIDLAVHSSKDMPTVLPDGLALTAFLPREDVRDAFLSTL
TDLPQGAVVGSSSLRRQAMIKRLRPDIRGNLQTRLRKLDATLLAAAGLRRLPAVGQGAICIESRADDIHDAATQIRLDAERAFLAVLDGSCRTP
IGGLAMHFRGAARIGRSVGEDL
>AP70_WP_150495167.1_Roseibium_aquae

RIGTRGSALALAQAHETRSRLEIVVIKTSGDQIQDRPLSGKGLFTKEIEDALLDGRIDLAVHSSKDMPTVLPGGLAITAFLPREDVRDAFLSTL
MDLPHGAVVGSSSLRRQAMIKRLRPDIRGNLQTRLRKLDATLLAHAGLRRLPAVGQGAICIESRIDDIDHLETKIRLTAERAFLAVLDGSCRTP
IGGLALTLKGAEAIGKAAGETL
>AP71_WP_074785829.1_Roseovarius_tolerans
KIGTRGSPLALAQAHETRARLEISVIKTTGDRIIDRPLKGKGLFTREIEEALLSGEIDIAVHSMKDMPVLQPEGLSLDTYLPREDVRDAFVSAL
ADLPAGARVGTSSLRRRAQVLVAYPHLRGNVQTRLKKLACTFLAMAGLNRLPAVAQGAIGIERRADDLHDTETGQRLAAERAFLEALDGSCETP
IAGLAVRLRGGAELGRAMAAKL
>AP72_TWI79925.1_Roseibium_hamelinense
RIGTRGSALALAQAHETRARLEIVVIKTTGDQIQDRPLSGKGLFTKEIEEALLDSRIDLAVHSSKDMPTVLPDGLALTAFLPREDVRDAFISTL
AGLPEGAVVGSSSLRRQAMIKRLRPDIRGNLQTRLRKLDATLLAYAGLKRLPAVGQGAICIESRVGDIHDQDTHVRLETERAFLAVLDGSCRTP
IGGLAIRFRGPEGLGRSAGEAL
>AP73_WP_095638908.1_Paracoccus_salipaludis
RIGTRGSALALAQAHETRDRLEIVVIKTTGDRVLDRPLRGKGLFTREIEDALLDQRIDIAVHSMKDMPVLQPEGLIIDCYLPREDVRDAFVSSI
AELPAGTLVGSSSLRRRAQLIHYRPDLRGNVQTRMRKLEATFLAMAGLTRLPAVAQGAIGVERRKDDISDLPTTLRVTAERAFLRRLDGSCETP
LAGLALWLRGGIEMGRDLAEEL
>AP74_WP_185663916.1_Novosphingobium_flavum
KLGTRRSPLAMAQAEEARARLELVPVTASGDRIQDRPLAGKALWTKELDAWLLAGEIDFAVHSLKDVETIRPAGIAIAAILPREDVRDVLVGSI
HALPRGARVGTSAPRRAAQLLHARPDCRGNVATRLAKLDATLLALAGLNRLPAPGQGAIAIECRAGDINDTPSHAAVTAERALLAALGGNCHSP
IAVLTLVMEADDAAPGRLADDL
>AP75_OYY76903.1_Sphingomonas_sp
RLGTRGSPLALTQARMVRDALEIVVIKTTGDQVQDRALAGKALWTKELDRAILAGEIDLAVHSMKDVETIRPEGIVISAMLPRADVRDRLIGSI
DALPQGATIGTSSPRRQAQLLRLRPDLRGNVDTRLAKVDATLLAAAGLDRLPAPAQGAVGIETRAADISHHPTMACVMAERALLLALNADCHSP
LAALALHLRADLGFAAQLAADL
>AP76_WP_184051858.1_Sphingobium_sp
RLGTRGSPLALAQAELTIAALRIVTVSTSGDRIQDRPLAGKALWTKELDRALLAGEIDCAVHSMKDVETIRPETITIAAMLPRAATQDRLIGSI
AAIPEGAVIGTSSPRRAAQMKALRPDLRGNVATRLARVDATLLAAAGLDRLPAVAQGAVGIECRADDIGDAATEACVRAERALLLALGGSCHSP
IAALAIRLRAPEAGAMTLGKEL
>AP77_WP_037498745.1_Sphingomonas_jaspsi
RLGTRGSPLALAQAHMVAAALAIHTVKTSGDRIQDRPLAGKALWTKELDVALLAGDTDISVHSMKDVESERPDILVIAAMLPRADVRDRLIGSI
AALAAGATVGTSSPRRAAQLRGKRPDLRGNVDTRLAKRDATLLAAAGLDRLPAPAQGAVGIECRGDDIDHRDTHAAVMAERAYTRALGGTCHSP
VAALAVDFRCDVDGPAALAREM
>AP79_NOU03564.1_Novosphingobium_sp
RLGTRRSPLAMAQAEETRARLVIVPVTASGDKVLDRPLAGKALWTKELDAWLLGGEIDIAVHSAKDVETERPDWLTIAAVLPREDVRDVLVGSL
AALPPGARIGTSAPRRAAQALHLRPDCRGNVQTRLAKLEVTLLAAAGLNRLPAPGQGAIAIECRTADIDHLPSRHAVMAERALLAALGGNCHSP
IAVLTLTMRADHAGPAALAADL
>AP82_WP_147089116.1_Novosphingobium_ginsenosidimutans
RLGTRRSPLAMAQAEEARDRLEIVPVQASGDKILDRPLAGKALWTKELDVWLLEGEIDFAVHSAKDVETLRPAGIAIAAVLPRADVRDVLVGSL
AALPPGARIGTSAPRRAAQALHFRPDCRGNVATRLGKLDVTLLAAAGLERLPAPGQGCIAIECRPGDIDHRASHAALLAERALLAALGGNCHSP
IAALTLVLHADATGPAELARLL
>AP83_WP_084573281.1_Erythrobacter_vulgaris
RLGTRRSPLAMAQAHEARARLELVPVVASGDKIQDRPLAGKALWTRELDIWLAEGKIDAAVHSMKDVETIRPAEIAIGAILPRADKADVLLGSL
SAIPQGARVGTSAPRRAAQLLHARPDLRGNVATRMAKLDATFLAAAGLARLPAPAQAAIGIECRVDDINHRPSAEEVLAERALLEGLGGTCHSP
IAALTLHMTADLDAARNLASEL
>AP84_WP_082134782.1_Croceicoccus_naphthovorans
RLGTRRSPLAMAQAYEARTRLEIVPVLASGDKVLDRPLAGKALWTKELDGWLIENRIDFSVHSMKDVETIRPEQIAIAALLPRADVRDVLVGSI
SQIPPGARVGTSAPRRAAQMLNLRPDLRGNVATRLAKLEVTLLAAAGLQRLPAPAQGAIGIECRVDDIDDAPTRAQVMAERALLLALGGNCHSP
IAVWTLTMTAKSDGPAELARQL
>AP85_WP_118054708.1_Altererythrobacter_sp
RLGTRNSPLAMAQAYETRQRLELVPVVASGDKVLDRPLAGKALWTKELDHWLHEGRIDASVHSMKDVETLRPETLTIAAVLPRADTADRLVGSV
ADIPEGATIGTSAPRRAAQLLHARPDCRGNVATRLSKLDCTLLASAGLDRLPAPAQGAIGIECRTDDINHAPSYQAVMAERALLKALGGNCHSP
IAMLTLALRCDLSWIEAMGEDL
>AP87_WP_138011819.1_Chakrabartia_godavariana
KLGTRASPLALAQAHMVRDALEIAAMTASGDRILDRPLAGKALWTKELDRALFEGEIDFAVHSMKDVETLRPAELAIVAMLPRADVRDRLIGSI
AALPQGARVGTSSPRRAAQLLRHRPDLRGNVATRLAKLDATLLAAAGLDRLPAPAQGAIGIETHAANINDAATFACVMAERAFLATLTADCHSP
VAAQALHIRG----PVALAQAL
>AP88_WP_100259400.1_Erythrobacter_seohaensis
RLGTRRSPLAMAQAEEARARLELVPVIASGDKIQDRPLAGKALWTRELDLWLVEGRIDAAVHSMKDVETIRPDEIAIGAILPRADKADVLLGSI
SAIPQGAKVGTSAPRRAAQLLHARPDLRGNVATRMAKLDATFLAAAGLSRLPAPAQAAIGIECRASDISHAESRDEVLAERALLQGLGGTCHSP
IAVLTLTMRADLAAPGELARTL
>AP89_WP_184197288.1_Polymorphobacter_multimanifer
KLGTRGSPLALAQANMTAAALEIVVINTKGDQVQDRALAGKALWTKELDAALLDGRIDCGVHSMKDVETQLRDGIALAAMLPRADVRERLLGSI
AALPQGAVIGTSSPRRAAQLKALRPDLRGNVATRMAKVDATLLAAAGLDRLPASAQGAVGITARLGDIDDADTHAAVLLERGFLAALGGSCHSP
VAALAVHFRAAADEVAALAVRL
>AP90_WP_082836126.1_Croceicoccus_sp
RLGTRSSPLAMAQAHEARTRLEIVPVMASGDKVLDRPLAGKALWTKELDGWLHEGLIDFAVHSMKDVETIRPDWLEIAALLPRADVRDVLVGSV
SAIPPGSRIGTSAPRRAAQMLNLRPDLRGNVATRLAKLDVTLLAAAGLERLPAPAQGAIGIECRRNDIDDGATRAQVMAERALLYALGGNCHSP
VAVWTLTMTAGPAGAAELAHKL
>AP91_WP_123154477.1_Aestuariisphingobium_litorale
KLGTRASPLALAQAHMVRDALEIVAMTASGDRILDRPLAGKALWTKELDRALFEGEIDFAVHSMKDVETLRPAELAIVAMLPRADVRDRLIGSI
AALPLSARVGTSSPRRTAQLLRHRPDLRGNVATRLAKLDATLLAAAGLDRLPAPAQGAIGIETHGANINDAATFACVMAERAFLATLSADCHSP
VAAQALHIRGPVAL----AQAL
>AP92_WP_160766511.1_Erythrobacter_citreus
RLGTRRSPLAMAQAHEARARLELVPVVASGDRIQDRPLAGKALWTRELDQWLVEGRIDGAVHSMKDVETLRPEAIAIAAILPRADKADRLLGAI
SDIPAGARIGTSAPRRAAQLLHLRPDLRGNVATRMAKLDATFLAAAGLDRLPAPAQAAIGIECRSGDIDDAPSHAEVAAERRLLEGLGGSCSSP
IAALSIALTADDAAPLALARGL
>AP93_WP_160729335.1_Altererythrobacter_luteolus

RLGTRQSPLAMAQAEETRRRLELVKVRASGDKIQDRPLAGKALWTRELDIWLAEGRIDAAVHSMKDVETLRPESLTIAAILPRADVRDVLIGSI
QSIPRGAVVGTSAPRRAAQLLHHRPDCRGNVATRLAKLQATFLAAAGLERLPAPAQGAIGIECNTDDINHQGSRDTVMAERALLEALGGTCHSP
IAVLSLTMKADLDAARTLGRDM
>AP94_WP_088306676.1_Novosphingobium_sp
RLGTRRSPLAMAQAEEAKARLEIVPVTASGDKIQDRPLAGKALWTKELDHWLLTGEIDFAVHSLKDVETLRPAELVIAAVLPREDVRDVLVGSI
RALPQGAVVGTSAPRRAAQLLHARPDLRGNVATRLAKLDATLLARAGLNRLPAPGQGAIAIECRLDAIDHTPSRHAVMAERALLAGLGGNCHSP
IAVLTLVMRADANGPAALAAQL
>AP99_WP_187449658.1_Caulobacter_sp
RIGTRASKLAQTQTRHMQARIPLVLITTTGDRIQDRRLLGKALFVKEIEEALLAGEIDVAIHSMKDVPAEQPAGLTIAAIPEREDPRDAFVSSL
AELPQGARLGTASLRRQAQTLADRPDLRGNVDTRLRKLDAILLAKAGLNRLPAPGQGALAIQSRVVDLHHAPTALAVAAERGALEALEGSCRTA
VGAHALVMTVARELGLDLGRQI
>AP100_WP_111527206.1_Phenylobacterium_sp
RIGARGSKLSLAQAGHMQRRIPLIVITTTGDRVQDRRLLGKGLFTKEIEEALLDGRIDCAVHSMKDMPADLPEGLCIAAIPEREDPRDAFVSRL
EDLPKGARLGTASLRRQAQSLHRRPDLRGNVDTRLAKLDAILLAYAGLRRLPAPGQGALAIETRISDLRDHPTTVAVAAERGALQALEGSCKTA
IGAHALKLIVARALGVSLGEAV
>AP101_WP_167175504.1_Brevundimonas_terrae
RIGTRRSKLALAQSGMMQRAIPLVEIVTTGDRIQDRRLLGKSLFTKEIEEALLEGRVDVAVHSMKDVPAEQPDGLALAAIPEREDPRDAFISSL
ADLPQGAKVGTASLRRQAQLLSIRPDLRGNVDTRLRRLDAILLACSGLNRLPAPGQGALALQTRASDLNHIPTALCVVAERGAMLALEGSCRTP
VGAFALHIGTITALGRQLGDAV
>AP103_OXE37704.1_Phenylobacterium_zucineum
RIGARGSKLSLAQAGHMQRAIPLIVITTTGDRVQDRRLLGKGLFTKEIEDALLSGEIDCAIHSLKDMPAACPPGLVIAAIPEREDPRDAFLSRL
EDLPQGARLGTASLRRQAQSLYRRPDLRGNVDTRLAKLDGILLALSGLKRLPAPGQGALAIQTRVEDLRHEPTVVAIAAERGALSALEGSCKTP
IGAHALHLIVARALGLELGAAV
>AP105_WP_183202760.1_Brevundimonas_lenta
LIGTRRSKLALTQSGMMQRAIRLVEIVTTGDQVQDRRLLGKALFTKEIEEALLAGRVDVAVHSMKDVPAEQPYGLAIAAVPEREDPRDAFVSSF
DELPMGARLGTASLRRQAQALALRPDLRGNVDTRLRKLDAILLAVSGLNRLPAPGQGALALQTRVEDLNHPLTALAVAAERGAMTALEGSCRTA
IGAHALRLTTARALGLRIGAEV
>AP106_MBA4095202.1_Rhodospirillum_sp
RIGTRGSPLALAQADEAHARLEICVIKTTGDQVQDRPLSGKGLFTKEIEEALVAGAIDIAVHSMKDVPTWLPDGLVIDCLLPREDPRDVLIASI
ATLPRGAVVGSASLRRAAQVKALRPDLRGNVETRIAKVTATLLALAGLKRLPAVAQGAIGLETRAGDLNDRNTQLRVAAERACLAVLDGSCRTP
IAAFAMRLRAAAALGIQIGRKL
>AP108_WP_012567533.1_Rhodospirillum_centenum
RIGTRGSPLALAQAHETRRRLEIVVIKTTGDRILDRTLMGKGLFTKEIEEALQAETIDLAVHSMKDVPTWRQTGLQLTAMLPREDPRDAWFCGL
DELPAGAVVGTASLRRQAQILARRPDLRGNVQTRMTKLDATLLALAGLRRLPAVAQGAIGIETRAADLNHAETMTRVTAERGVLATLDGSCRTP
IAALALTLSAAEAMGIDAGQEL
>GP2_WP_041406288.1_Shewanella_loihica
RIATRKSPLAMWQAEFVKAELELLPMSTKGDVILDTPLAGKGLFVKELEVAMLEGKADIAVHSMKDVPVDFPEGLGLQVICEREDPRDAFVSNI
EELPQGAIVGTSSLRRQCQIRAARPDLRGNVGTRLAKLDAIILAAAGLKRLPANGQGAVGIECRTDDLEHIETRYRVIAERAMNTRLEGGCQVP
IGAYALSLRGAEQLGIALADEL
>GP3_WP_153858699.1_Serratia_proteamaculans
RIATRQSPLALWQAHYVQQRLELVPMVTKGDIILDTPLAGKGLFVKELELALLDGRADIAVHSMKDVPVDFPAGLGLVTICERDDPRDAFVSSL
DQLPQGSVVGTSSLRRQCQLRERRPDLRGNVGTRLSKLDAIILAVAGLKRLPAVGQGAVGIECRLDDLNHAVTETRVRAERAMNTRLEGGCQVP
IGSYALWLRAAEQMGIELAEEL
>GP4_WP_125868777.1_Pseudomonas_stutzeri
RIATRKSALALWQAEYVKARLSLVPMVSRGDKLLDAPLAGKGLFVKELETALLENEADIAVHSMKDVPMEFPEGLGLYCICEREDPRDAFVSSL
DALPQGSVVGTSSLRRQAQLLARRPDLRGNVNTRLAKLDAIILAAAGLIRLPAGGQGAVGIECRTGDLNHAPTATRVVAERALNKRLNGGCQVP
IACYALWLRGAEALGVQVAEEL
>GP5_WP_073262886.1_Pseudomonas_punonensis
RIATRKSALALWQAEHVKARLSLVPMVSRGDKLLDAPLAGKGLFVKELETALLENQADIAVHSMKDVPMDFPEGLGLFCICEREDPRDAFVSSL
DALPAGSVVGTSSLRRQAQLLARRPDLRGNVNTRLAKLDAIILAAAGLIRLPAGGQGAVGIECRVGDLNHTETALRVTAERAMNKRLNGGCQVP
IACYALWLRGAEGLGIEVAEAL
>GP6_WP_120134465.1_Rahnella_woolbedingensis
RIATRQSPLALWQAQYVQSQLELVPMVTKGDIILDTPLAGKGLFVKELELAMLDGRADIAVHSMKDVPVEFPEGLGLVTICEREDPRDAFVSSL
AELPHGSVVGTSSLRRQCQLRQKRPDLRGNVGTRLSKLDAIILAVAGLKRLPAVGQGAVGIECRLDDLAHRETTVRVEAERAMNMRLEGGCQVP
IGSYALWLRAAHSMGVELAEEL
>GP7_WP_158651152.1_Aeromonas_veronii
KIATRKSPLALWQANFVKDRLELVPMSTQGDKILDTPLAGKGLFVKELETAMLEGRADIAVHSMKDVPVEFPAGLGLHTICEREDPRDAFVSAI
AELPQGAVVGTSSLRRQCQLRAARPDLRGNVNTRLAKLDAIILAAAGLKRLPANGQGAVGIECRLDDLEHAETRARVLTERAMNRALQGGCQVP
IGAYAIWLRGGEALGEQLAQRL
>GP9_ACB16089.1_Escherichia_coli
RIATRQSPLALWQAHYVKDKLELVPMVTRGDVILDTPLAGKGLFVKELEVALLENRADIAVHSMKDVPVEFPQGLGLVTICEREDPRDAFVSSL
DALPAGSIVGTSSLRRQCQLAERRPDLRGNVGTRLSKLDAIILAVAGLKRLPAVGQGAVGIECRLDDLNHHETALRVTAERAMNTRLEGGCQVP
IGSYAIWLRAAEQMGISLAEEL
>BP1_SQF93518.1_Paucimonas_lemoignei
RIATRKSALALWQAEYVKGRLTLVPMVSRGDKLLDSPLSGKGLFVKELETALLEDRADIAVHSMKDVPMDFPQGLGLFCICEREDPRDAFVSSL
EALPAGSVVGTSSLRRQAQLLARRPDLRGNVNTRLAKLDAIILAAAGLIRLPAGGQGAVGIECRSVDLHHDDTATRVTAERSLNKHLNGGCQVP
IACYAIWLRGAQALGVQVAESL
>BP2_MBC7672458.1_Polaromonas_sp
RIGTRTSELALRQARMVQAALELVTYKTVGDKRLDESLSAKGLFTKELETGLLKRKIDVAVHSLKDLPTESPHGLVVAAVLEREDPRDALVVSL
DGLPRGSRVGTSSLRRRAQLLATRPDLRGNVPTRLKKVHAAILAAAGLHRLPAAGQGAIAVQTRLDDMNDAPTMRAVRAERAFLAALEGGCQVP
IGALAAMLHGPELSGIRLANQL
>BP3_WP_136771940.1_Chitiniphilus_eburneus
VIATRESPLALWQAEHVRAWLELLGMTTQGDRILDVTLNGKGLFVKELEQALADGRADLAVHSMKDVPMVMPDGFALTAIGEREDPRDALVSTL
EQLPEGAVVGTSSLRRQEAQLRARFPHLRGNVGTRLAKLDAILLASAGLKRLPAPGQGALGLEIRADRFNHPATAAAVTAERALSRRMGGSCQIP
LAAYALRLRGAEGLGLKVAELL
>BP4_WP_007881387.1_Herbaspirillum_sp

VIASRESRLAMWQAEHVRDRLIILGMTTRGDQILDRTLSGKGLFVKELEVAMAEGRADFAVHSLKDVPMELPQGFVLGGILEREDPRDAFVSGL
DDLPHGAVVGTSSLRRQALIAARYPHLRGNLDTRLAKLAAIILAVAGLKRLPAPGQGAMAIEILADRINHLPTDRAVTAERTLSRTFGGSCQIP
LAAFAMHMRAPEALGRQVADLL
>BP5_WP_038490856.1_Collimonas_arenae
VIASRESRLAMWQAEHVRARLEILGMTTRGDQILDRTLSGKGLFVKELEVAMVEGRADLAVHSLKDMPMDLPSGFALAAVLEREDPRDAFVSSL
AELPAGAVVGTSSLRRQALIAARFPLLRGNLDTRLAKLAAIILAAAGLKRLPAPGQGAMAIEICDDRLNHLPTEQAVTAERTLSRAFGGSCQIP
LAAFAMRLRAPQALGGKIAQDL
>BP6_KAB2868486.1_Burkholderiaceae_bacterium
RIATRQSALALWQAEHVAARLELVPMTTRGDQILDRPLAGKGLFLKELEVALQQGRADIAVHSLKDVPMTLEPGFAIAAVLERADAADAFVSEL
AQLPRRARVGTSSLRRQAQLRALRPDLRGNVNTRLARLDAIVLACAGLERLCAVGQGAIAIECRTDDLDDATTRRCTDAERAMSLALHGSCNVP
IAAYALSLWGAQALGQRVAQLL
>BP7_WP_082814661.1_Collimonas_fungivorans
VIASRESPLAMWQAEHVRERLEILGMTTRGDQILDRTLSGKGLFIKELEVAMEEGRADLAVHSLKDMPMELPPGFVLSAVLEREDPRDAFVSSL
EQLPAGAIVGTSSLRRQALIAARFPLLRGNLGTRLAKLAAIILAAAGLKRLPAPGQGAMAIEIRADRLNHLPTELAVTAERTLSRAFGGSCQVP
LAAFAMRLRAPEVLGETIAAAL
>BP8_WP_115432742.1_Crenobacter_cavernae
VIASRESRLAMWQAEHIKARLSILGMTTQGDQILDKTLSGKGLFVKELEVALAEGRADLAVHSIKDVPMALPEGFALAAICEREDPRDAFVSSL
AELPDGAVVGTSSLRRESQLRARYPHLRGNVQTRLAKLDAIILAAAGLKRLPAPGQGALGIEIRADRLDHADTRDCVLAERALSRELGGSCQVP
LGAYAVTLGGADALGRAVAKKL
>BP9_SAH99349.1_Bordetella_trematum
VIATRASRLALWQAEHVRDRLELLTLTTRGDQILDRTLSGKGLFVKELEHALLDGRADLAVHSLKDVPVDMHAPFALSTILERADCRDAFVSSL
DELPAGAVVGTSSLRREAQIRAARPELRGNLDTRLGKLDAVVLAAAGLQRLPAAGQGALGIEIRDDRLACAHTTACVSAERAVSRTLGGSCQVP
LAAFALQIDAAERLGIEAAQEL
>BP11_WP_173143141.1_Achromobacter_pestifer
VIATRASRLALWQAEHVRDRLELLTLTTRGDQILDRTLSGKGLFVKELENALLDGRADLAVHSLKDVPVDLQAPFELCAVLDRADPRDAFVSSL
ADLPAGAVVGTSSLRRESQIRARYPELRGNLDTRLGKLDAIVLAAAGLERLPAAGQGALGIEIRDDRLVSADTTSCVVAERAVSRKLGGSCQVP
LAAYALSLRAAQAIGEAAAQAL
>EP2_WP_122873789.1_Campylobacter_showae
KIATRKSVLAMWQSEHIRDKIELTGMKTKGDVILDTPLAGKGLFTKELEDSMLSGETHIAVHSLKDVPVVFPDGLVLAAICSREDVRDAMLSKF
EDLPQGARVGTTSLRRKMQLLAMRPDLRGNVQTRLRKLDAIILAMAGVNRLPAMGQGALGIEAR-
EDLKDEKAVIETTVERDFVAMLEGGCQVPIGVNAIEIRAWKSVGAELGRIF
>EP3_WP_084041450.1_Campylobacter_concisus
KIATRKSILALWQSEHIKARIELIGMKTKGDVILDTPLAGKGLFTKELEDSMLKGETDIAVHSLKDVPVVFPEGLKLAAICSREDTRDAMISKF
SDLPHGAKVGTTSLRRKMQLLIMRPDLRGNVQTRLRKLDAIILAMAGINRLPAMGQGALGVEAR-
DELNDENAVIETTIERDFVSVLEGGCQVPIGISAISIDAFQSVGKELAHKF
>EP4_WP_169780061.1_Campylobacter_curvus
KIATRKSVLALWQSEHIKARLELVGMKTKGDVILDTPLAGKGLFTKELEDSMLSGQTHIAVHSLKDVPVAFPDGLVLAAICSREDVRDAMISKF
DDLPQGAKVGTTSLRRKMQLLRMRPDLRGNVQTRLRKLDAIILAMAGINRLPAMGQGALGVEA-
VDDLNDERSVIETRVERDFVTLLEGGCQVPIGINAIEINAFESVGKELAREF
>EP5_WP_103645015.1_Campylobacter_concisus
KIATRKSILALWQSEHIKARIVLEGMKTKGDVILDTPLAGKGLFTKELEDSMLKGETDIAVHSLKDVPVVFPKGLKLAAICSREDTRDAMISKF
SNLPHGAKVGTTSLRRKMQLLIMRPDLRGNVQTRLRKLDAIILAMAGINRLPAMGQGALGIEAR-
DELNDENAVIETTIERDFVSVLEGGCQVPIGISAISIDAFQSVGKELAHKF
>DP1_WP_155874870.1_Desulfuromonas_sp
RIGTRASQLALWQANWVKAELTLTKIKTQGDKILDVPLAGKGLFVKEIEEAMLRGEIDIAVHSMKDVPTIFPEGLALRCITKREDPRDICVISF
RDLPQGARIGTSSLRRKAQLLHLRPDFRGNVETRIRKLHAVILAAAGMHRLPAIGQGALGLESRLDDFNHPETAAAVIAERALLRRLEGGCQVP
IAAYGLTFTGAEKIGISVADDL
>DP3_WP_092057636.1_Desulfuromonas_acetexigens
RIGTRASQLALWQANWVKSELTLTKIKTQGDKILDVPLAGKGLFVKEIEEAMLRGEIDIAVHSMKDVPTFFPEGLALRCITEREDPRDIVVLSF
RDLPQGARIGTSSLRRKAMLLHLRPDFRGNVETRIRKLDAVVLAAAGMHRLPAIGQGALGIESRIADFNHPETAAAVKGERAVLRRLEGGCQVP
IGAYGLTLTGSEKVGISLADDL
>DP4_WP_067618923.1_Dissulfuribacter_thermophilus
RLGTRKSKLALTQSNWVKQEIELVKVTTKGDKILDVPLAGKGLFVKEIEEALLDGRIDFAVHSLKDVPTELPQGLEVSVFPEREDPRDALIAGL
MELPPGAKVGTSSLRRMAQLRAVRPDLRGNLDTRLNKLDAIILAAAGLKRMPAIGQGALGIEFRSEDLSHEETTIRVRAERAFLARLEGGCQVP
IGAYALTLEGPEFIGDELGKEM
>DP5_RQW86780.1_Geobacter_sp
KIGTRSSPLALWQAEWVSARITLVKIKTTGDMILNVPLAGKGLFVKEIEEAMLRGEVDIAVHSMKDVPTVFPPGLGLTCISKREDPRDALVSSF
AELPHGARVGTSALRRQAQLLAHRPDLRGNVQTRLAKLDAVILAAAGLKRLPAIGQGALGIECRLDDLNDLETQRAVGAERAFLLRCEGGCQVP
IAAFALRLTGFLSLGTELADRL
>DP6_WP_012646388.1_Geobacter_daltonii
RIGTRASQLALWQANWVKSELSLVKIKTIGDKILDVPLAGKGLFVKEIEEAMLRGDIDIAVHSMKDVPTEFPEGLGLHCITEREDPRDAVISKF
ADLPQGAKIGTSALRRQAQLLKVRPDMRGNVETRINKLDAVILAAAGLKRLPAIGQGALGIECRLDNFNHPATAYAVRAERALLWRCEGGCQVP
IAAFGLKLVGCEKLGIKLAEQL
>DP7_WP_040199015.1_Geoalkalibacter_subterraneus
RIGTRSSQLALWQANWVKNCLTLTKIKTQGDKILDVPLAGKGLFVKEIEEAMLRGEVDIAVHSMKDVPTIFPEGLDLRCITEREDPRDIVVLSW
RDIPQGGRIGTSSLRRKSQLLHLRPDLRGNVETRIRKLDAVVLAAAGMKRLPAIGQGALGLETRIDDFHHPETAYAVTAERALLRHLEGGCQVP
IAAYGVNLTGAAQLGISLAEDL
>DP8_WP_119333830.1_Geobacter_sulfurreducens
RIGTRASQLALWQANWVKSELELVKIKTIGDKILDVPLAGKGLFVKEIEEAMLRGEIDIAVHSMKDVPTEFPEGLGLVCITEREDPRDAFISTF
ADLPQGAKIGTSALRRQAQLLKVRPDLRGNVETRIRKLDAVILAAAGLKRLPAIGQGALGLECRLDDFNHPDTAHAVRAERALLWRCEGGCQVP
IAAHGLTLTGCEKLGIALAEKL
>DP9_WP_022660166.1_Desulfovibrio_desulfuricans
VIATRGSQLALWQAEHVKSRLDLVIIKTKGDIIQDVPLAGKGLFVKEIEEALLDNRADLAVHSIKDVPMELPDGLILGCIPKREAPTDCMLSDL
AALPQGACVGTSSLRRQAQLLALRPDLRGNVDTRLRKLDAIILASAGMNRLPAVGQGAIGIECREDDIEDTPTRVCVEAERGFLAGLEGGCQVP
IAGHAFVLEGARQIGLELAEKL
>DP10_WP_077072293.1_Mailhella_massiliensis

IIATRGSKLALWQAEHVRERLELLVLKTKGDIILDVPLAGKGLFVKEIEDALLTGAADIAVHSMKDVPMVLPEGLILGAVPEREVCNDLFLSSL
ADLPSGAVLGTSSLRRQAQVLAMRPDLRGNVETRLRKMDAIILARAGVKRLPAVGQGALGVEMREDRMEDEPTRLCVMAERSFLRRLDGGCQVP
IAAHALSLQAAERMGVALAEDL
>DP11_WP_066801385.1_Pseudodesulfovibrio_indicus
TIATRGSALALWQANHIKDRLDLLKIKTKGDKILDVPLAGKGLFVKEIEEALLDGRAQLAVHSMKDVPTELPEGLEVGIIPEREAHTDTLCSGL
KDLPRGAVVGTSSLRRQSQIMALRPDLRGNVGTRVQKLDAIILATAGLKRLPAVAQGALGIEYRIDDLDHPATKHQVLAERGFLTGLDGGCQVP
IAAWSVRLTGAWDVGMVLAGKV
>F4_WP_007506293.1_Caldalkalibacillus_thermarum
VLGSRRSKLALTQTRWVIRQLEIKEIVTKGDKIVDRMLSGKGLFVKEIEQALLEGEIDFAVHSVKDMPAELPPGLELACVPKREDPRDCLISTL
DSLPDGAVIGTSSLRRAAQILHARPDVRGNVDTRLRKLDAIILAASGLKRIPAVGQGALGIECRASDIHHEPSGLAVMAERAFLAEMGGSCHVP
VACYALVLTGPVQLGRQAAQRL
>F5_WP_094236160.1_Tumebacillus_algifaecis
VVGTRKSPLALTQTEMVMAKLGMEKIVTKGDRILDVTLSGKGLFIKEIEQAMYEKKIDFAVHSLKDMPTEMPEGLMLAAITEREDPRDVVITTF
ADLPIGARVGTSSLRRAAQLLNARPDIRGNINTRIAKLDAIVLAAAGLERMPAVGQGALAIQCRSEDLDDVNTRRTVTAERTVLGRLNGGCQIP
IGAFALKLTGPMALGHAVAERL
>F7_WP_033099238.1_Thermoactinomyces_daqus
IVGTRKSQLAMTQTSWVVDRLRMEKIVTKGDRILNVTLSGKGLFVKEIEQALLDGRIDFAVHSMKDLPGEMPEGLVIGAIPRRENPRDVLLSML
AELPPGSVVGTSSLRRQAQILASRPDLRGNLDTRIRKMDAIVLAAAGLYRMPAVGQGALAIQCREDDINHEQTARAVRAERAFLVAFEGSCHMP
LAAYAVHLTGEWAVGKQLAKEM
>F10_WP_048355980.1_Bacillus_glycinifermentans
IVGSRRSKLAMTQTKWVIKKLEIKEIVTKGDRILDVTLSGKGLFVKEIEQAMLSGEIDMAVHSMKDMPSALPESLVIGCVPKREDVRDALISTL
AELPQGAVVGTSSLRRSAQLLQIRPDLRGNIDTRLKKLDAIILAAAGLSRMPAVGQGALAIECRAADLNDCYTEQTVTAERAFLHAMEGGCQIP
IAGFAIELTGPAAVGKECAERM
>A5_SGC95152.1_Mycobacterium_tuberculosis
RIATRQSPLALWQAEFVRQELELVPMVTKGDIILDTPLAGKGLFVKELELALLENRADIAVHSMKDVPVDFPDGLGLVTICEREDPRDAFVSSV
DALPPGSIVGTSSLRRQCQLRERRPDLRGNVGTRLGKLDAIILAVAGLKRLPAVGQGAVGIECRLDDLNHAQTDICVRAERAMNTRLEGGCQVP
IGSYAIWLRAAAAAGVSLAEEL
>A7_WP_066890085.1_Streptomyces_thermoautotrophicus
KLGTRKSSLAMAQARAVAEAIELVGITTYGDTTK-
AALAGTGVFVNALRDALLEGRIDLAVHSLKDLPTTQPDGLVIAAVPPREDPRDALCAKLTELSAGARVGTGSPRRAAQLRALGYGLRGNVDTRL
RYVDAVVLARAGLARLPAPGQGALAVECRAGHLDDPDTRAAVTAERALLAALEAGCSAPVGAFGLYLRGADQLGRRLAAEM
>A8_WP_073262358.1_Cryptosporangium_aurantiacum
RLGTRASKLALAQSGDVAAALELVHISTDGDRDRTTPLTGTGVFVSALRDALLADEIDFAVHSYKDLPTAAAEGLTIAAVPVRADTRDALIATL
EKLPAGARIGTGAPRRIAQLLAARPDVRGNVDTRLGKVDAVVLAVAGLTRLPAPAQGALAIECRADDLEDSGARAEVVAERTLLATLEAGCSAP
VAAWALTLRAAETVGRQLAADL
>A9_WP_110240992.1_Nocardioides_gilvus
RLGTRASALATTQSGMVADAIELVHITTEGDVNR-
GPLAGTGVFVSALRDALLDGRVDLAVHSLKDIPTTPAEGITLAAVPQREDPRDVLVATLGELPAGSKVGTGSPRRAAQLAALGLGLRGNVGTRI
GKVDGILLAHAGLNRLPAPGQGALAIECRTEDLDHAPTRAAVVCERQVLATLEAGCSAPLGALALWIRAAREIGQRLGSEM
>BA2_WP_159761666.1_Chryseobacterium_sp
RIATRQSPLALWQAHYVKDRLELVPMVTRGDVILDTPLAGKGLFVKELELALLEDRADIAVHSMKDVPVEFPPGLGLTTICERDDPRDAFVSSI
DALPAGCVVGTSSLRRQCQLAADRPDLRGNVGTRLGKLDAIILAVAGLKRLPAVGQGAVGIECRLDDLNHADTAVRVCAERAMNTRLEGGCQVP
IGSYAIWLRAAKELGISLAEEL
>BA3_WP_159612286.1_Tenacibaculum_sp
RIATRKSALALWQAEYVKAKLELVPMSTQGDKILDTPLAGKGLFIKELEIAMLEGRADIAVHSMKDVPVEFPDGFGLHAICERENPFDAFVSSL
DALPQGAVVGTSSLRRQCQIRKYRPDLRGNVNTRLAKLDAIILASAGLIRLPAVGQGAVGIECRNDDLNHTDTNTRVTAERAMNERLEGGCQVP
IGSFALTLTGAKDIGIEVAEAL
>BA5_OOK62944.1_Pedobacter_himalayensis
RIATRQSPLALWQAHYVKQRLELVPMVTRGDVILDTPLAGKGLFVKELELALLENRADIAVHSMKDVPVEFPEGLGLVTICEREDPRDAFVSSL
DALPAGSVVGTSSLRRQCQLAERRPDLRGNVGTRLGKLDAIILAVAGLKRLPAVGQGAVGIECRMVDLNHDDTAVRVKAERAMNTRLEGGCQVP
IGSYALWLRAAEALGVSLAEEL
>BA7_WP_171572752.1_Flavobacterium_sp
RIGSRKSQLALVQTYWVKDCLEVHTMSTQGDKILDVALADKGLFTKELEVGMITGDIDFAVHSLKDLPTNLPDGLMLGCVTERENPADALVVQL
DTLPEGAVIGTSSLRRLAQLRHHFPHLRGNLNTRLAKLDGLILAVAGLKRLHAVGQGALGIECRVGDLEHLPTAYRCYAERSFLRELEGGCQVP
IGVNTLTLTGADQIGIQLAQQL
>BA8_WP_106151552.1_Marinilabilia_salmonicolor
NIGTRSSQLAMWQAEAVKKALEIVPINTKGDAVLDVALSDKGLFTKEIENALLDGRVDVAVHSLKDLPTLLPEGLTLGGVLPRGEVRDVLVSGL
NELTSADKIGTSSLRRQAQLLHFNPELRGNVNTRLRKMDAMIMAGAGFIRLPAVSQGAVAMEIRDNDITDPKSLLTTSAERVFLKTIEGGCQVP
VACYSITLTGANANARELGQKL
>BA10_WP_011404301.1_Salinibacter_ruber
VLGTRGSELALRQANVVRSRLELETITTRGDEATDTPISDEAVFTKELDRALLQGGVDLAVHSLKDIPSTVPSGLALAAIGEREDPRDAFVAHL
HDLPEGATVATASLRRTAQLLTWRSDLRGNVDTRLEKLTGMVLAVAGLVRLPAVGQGALGIACREDQLHHAPSGHAANAERAFLKKVGGGCQVP
LGAWALVLDAGAAVGRKLAADL
>BA11_WP_018360336.1_Porphyromonas_macacae
IVGTRGSKLALAQTELLCQALETKIIKTKGDRFLELSLSDKGLFTREIEAQLLDGAIDMAVHSLKDLPVACVPGTMIGAYLERAKTEDVLIGTL
EELPEGAVVGTSSKRRAFQLSEKYPHFRGNVETRIAKMDAILMARAGMERLPAPGQAAIAIHIRNNDIDHKTTRYEVEFERELLQSLGGGCAMP
LGCVCNEVEVREATMKKLVMEL
>BA12_HEK45059.1_Rhodothermus_marinus
RLGTRGSLLARQQALSICAFLQVVIIQTTGDQIQEVPLASKGLFTKELDQALLEGRIDLAVHSLKDLPTELPKGLVLAAIPERASPWDVFVATL
ADLPTGAVLATSSLRRQAQLRAWRADVRGNVDTRLRRLHGLILAEAGLMRLPAVGQGALGVVCNADNMHHPPTAAAVRAERAFLRALEGGCQVP
VAALGLVLRGPEALGEALAAEL
